# A nationwide survey of awareness and understanding of NUT carcinoma among clinicians in China

**DOI:** 10.1186/s12913-026-14236-4

**Published:** 2026-03-02

**Authors:** Zhuomiao Ye, Dan Yang, Weiying Liu, Xin Li, Minghui Zhang, Mingzhu Yin

**Affiliations:** 1 Clinical Research Center (CRC), Chongqing University Three Gorges Hospital, Chongqing University, Wanzhou, Chongqing, China; 2 Cancer Early Detection and Treatment Center (CEDTC), Chongqing University Three Gorges Hospital, Chongqing University, Wanzhou, Chongqing, China; 3 Medical Pathology Center (MPC), Chongqing University Three Gorges Hospital, Chongqing University, Wanzhou, Chongqing, China; 4 Translational Medicine Research Center (TMRC), Chongqing University Three Gorges Hospital, Chongqing University, Wanzhou, Chongqing, China; 5 Institute of Advanced Interdisciplinary Studies, Chongqing University, Chongqing, China; 6 Chongqing University Three Gorges Hospital & Academy for Advanced Interdisciplinary Technology, CQU - Ferenc Krausz Nobel Laureate Scientific, Chongqing, China

**Keywords:** NUT carcinoma, Rare tumors, Clinician awareness, Survey, Oncology education

## Abstract

**Background:**

NUT carcinoma is a rare and highly aggressive malignancy defined by *NUTM1* gene rearrangement and is frequently misdiagnosed because of non-specific clinicopathological features. We assessed clinicians’ awareness and understanding of NUT carcinoma in China.

**Methods:**

We conducted a nationwide cross-sectional online survey (December 29, 2023–March 27, 2024) distributed via Wenjuanxing and professional networks. Eligible respondents were physicians and in-service trainees practicing in China. Responses with unclassifiable key information (e.g. city/department) were excluded. The questionnaire assessed self-reported awareness across five domains (clinical manifestations, diagnostic approaches, treatment, prognosis, and common *NUTM1* fusion partners) and awareness of NUT carcinoma–related organizations. Descriptive statistics were reported as n (%). Multivariable ridge logistic regression with five-fold cross-validation was used to explore associated factors, with Bonferroni correction for multiple comparisons.

**Results:**

Among 2222 returned questionnaires, 7 were excluded, leaving 2215 for analysis; 81.2% of respondents were from tertiary hospitals. Overall awareness was low: 1445 (65.2%) reported no awareness of any domain, and awareness rates were 631 (28.5%) for clinical manifestations, 552 (24.9%) for diagnostic approaches, 417 (18.8%) for treatment, 478 (21.6%) for prognosis, and 214 (9.7%) for *NUTM1* fusion partners. Only a small minority reported awareness across all assessed domains (approximately 7%). Awareness of related organizations was also limited: 1523 (68.8%) reported knowing none; 664 (30.0%) knew the “NUT Carcinoma Genetic Diagnosis Working Group” of the Chinese Anti-Cancer Association, 224 (10.1%) knew the NUT Carcinoma Registry, and 214 (9.7%) knew the European Cooperative Study Group for Pediatric Rare Tumors. In multivariable models, oncology and pathology were associated with higher awareness, particularly for diagnostic approaches (oncology OR 2.04, 95% CI 1.67–2.51; pathology OR 9.38, 95% CI 6.40–13.21) and prognosis (oncology OR 2.22, 95% CI 1.82–2.76; pathology OR 4.71, 95% CI 2.95–7.22), and with lower odds of reporting “no awareness” (oncology OR 0.52, 95% CI 0.43–0.63; pathology OR 0.15, 95% CI 0.10–0.21) (all Bonferroni-corrected *p* < 0.001).

**Conclusion:**

Most surveyed clinicians reported limited awareness of NUT carcinoma, including in tertiary hospitals. Practical strategies should prioritize training for pathology services and tumor-related specialties and strengthen referral pathways and access to NUT immunohistochemistry and molecular diagnostics to reduce missed or delayed diagnoses.

**Supplementary Information:**

The online version contains supplementary material available at 10.1186/s12913-026-14236-4.

## Introduction

NUT carcinoma is a rare and highly aggressive malignancy defined by rearrangement of the *NUTM1* gene, most frequently arising in the thoracic cavity and head and neck regions [1]. Despite advances in molecular diagnostics, the prognosis for patients remains extremely poor, with a median overall survival of just a few months [2, 3]. The clinical and pathological features of NUT carcinoma are often non-specific, leading to frequent misdiagnosis as other poorly differentiated cancers or sarcomas. Although several *NUTM1* fusion partners such as *BRD4*, *BRD3*, and members of the zinc finger protein family have been identified, there remains considerable uncertainty regarding optimal management, as current treatment strategies are largely extrapolated from protocols for other tumor types at similar sites [4–6].

One of the major challenges in improving outcomes for NUT carcinoma is the widespread lack of awareness among healthcare professionals [4]. Globally, and particularly in Asia, clinical recognition of this entity remains limited. This is reflected in the fact that international clinical registries include very few cases from Asian countries, and most published studies are limited to case reports or small series. In China, the situation varies considerably across institutions: while specialized pathology and oncology centers may have greater familiarity with NUT carcinoma, it is often not considered in the differential diagnosis of poorly differentiated tumors at many hospitals, and the use of specific diagnostic tools such as NUT immunohistochemistry is not yet routine in most settings. Consequently, many patients may not be accurately diagnosed until late in the disease course, potentially missing the opportunity for timely and appropriate management.

Given these challenges, it is critical to assess the current level of awareness about NUT carcinoma among Chinese clinicians. While definitive diagnosis relies on pathology and molecular confirmation, awareness among tumor-related clinical specialties can influence whether appropriate biopsies are obtained, whether NUT-specific testing is requested, and whether patients are referred to equipped centers. To address this gap, we conducted a nationwide survey aimed at evaluating clinicians’ familiarity with NUT carcinoma across different specialties and regions.

## Methods

### Development of knowledge questions on rare diseases

A nationwide cross-sectional survey was conducted in 2024 among clinicians from various provinces and hospital levels across China. Hospital levels were collected according to the Chinese hospital classification system (tertiary A/B/C, secondary A/B/C, primary) and harmonized into three categories (primary, secondary, and tertiary) for analysis due to small cell sizes in some subgrades. Participants were recruited via professional societies and online platforms. The questionnaire comprised sections on demographic and professional background, knowledge of NUT carcinoma (including definition, features, diagnosis, and management), and experience with suspected or confirmed NUT carcinoma cases. The full list of survey questions is provided in Additional file 1. The survey was distributed using the Wenjuanxing platform (https://www.wjx.cn/) and disseminated through social media (WeChat) as well as the Chinese Anti-Cancer Association (CACA) to reach physicians and in-service trainees nationwide.

Given the descriptive nature of this study, no formal sample size calculation was performed. The aim was to capture a broad spectrum of clinicians from diverse geographic regions and clinical backgrounds to assess overall awareness of NUT carcinoma in China. The survey was not restricted to any specific medical specialty. As the target population comprised only healthcare professionals, patients and the general public were not involved in the questionnaire development. No personally identifiable information was collected, and non-respondents could not be specifically identified. Responses were collected between December 29, 2023, and March 27, 2024. Eligible respondents were clinicians (physicians and in-service trainees) of any specialty and hospital level currently practicing in China. The questionnaire assessed self-reported awareness rather than verified knowledge. The only exclusion criterion was responses with unclassifiable key information (e.g., city or department). We note that a small subset of respondents selected ‘Administration/Logistics’ as their department; this may reflect clinicians with administrative duties.

## Statistical analysis

After downloading and organizing the collected responses, descriptive statistics were performed to summarize participant characteristics, with categorical variables presented as frequencies and percentages. To evaluate the association of variables such as city, specialty, and hospital level with NUT carcinoma awareness, multivariable ridge logistic regression models were applied. Hospital levels were recoded according to Chinese hospital classification standards (primary, secondary, and tertiary). The optimal penalty parameter (lambda) was selected via five-fold cross-validation, and bootstrap resampling (*R* = 200) was conducted to calculate standard errors and confidence intervals. All regression results are reported as odds ratios (ORs) with 95% confidence intervals. The Bonferroni method was used to adjust significance levels for multiple comparisons. Model performance was evaluated using area under the receiver operating characteristic curve (AUC) for discrimination and Brier score for predictive accuracy. Variance inflation factors (VIFs) were calculated to assess multicollinearity. All statistical analyses were performed using R version 4.5. Technical details regarding software packages and extended diagnostics are provided in the Supplementary Methods.

## Results

**Fig. 1:**
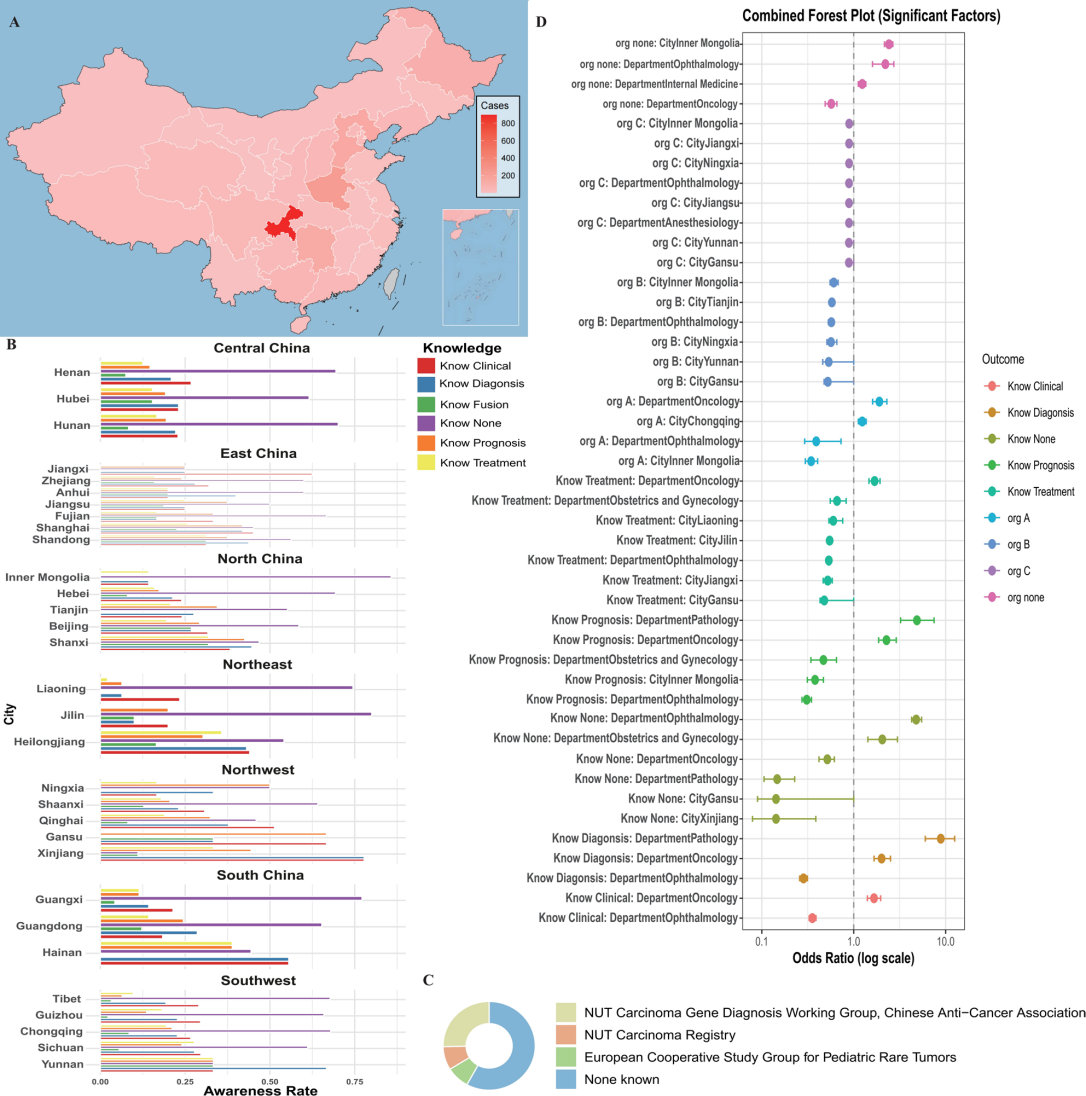
Geographic distribution, awareness, and statistical analysis of NUT carcinoma knowledge among Chinese clinicians. (**A**) Geographic distribution and sample size of survey respondents across 31 provinces, autonomous regions, and municipalities in China. The color gradient reflects the number of respondents per region, with higher concentrations in Chongqing, Henan, Heilongjiang, Hebei, and Hunan, and relatively fewer participants from Qinghai, Hainan, Ningxia, and Jilin. (**B**) Awareness rates of NUT carcinoma knowledge (clinical manifestations, diagnosis, fusion gene, prognosis, treatment, and “none known”) by province. The “Know none” category indicates respondents with no knowledge of any aspect. (**C**) Awareness of relevant professional organizations related to NUT carcinoma among respondents. The pie chart displays the proportion of clinicians aware of the NUT carcinoma gene diagnosis Working group (Chinese Anti-Cancer Association), the NUT carcinoma Registry, the European Cooperative study group for Pediatric rare tumors, and those with no knowledge of any organization. (**D**) Combined forest plot of significant factors associated with NUT carcinoma knowledge, as identified by multivariable ridge regression. Odds ratios (log scale) are shown for key variables, including department, city, and awareness of professional organizations, for each knowledge outcome (clinical, diagnosis, fusion, prognosis, treatment, and “none known”). Error bars indicate 95% confidence intervals

This survey covered 31 provinces (including autonomous regions and municipalities) across China (Fig. 1A), yielding a total of 2222 responses. Seven responses were excluded due to unclassifiable department or city information. According to backend statistics, the average completion time for the questionnaire was 77 seconds. According to the 2023 China Health and Wellness Statistical Bulletin, there were approximately 5.05 million registered physicians and assistant physicians (the two categories of licensed medical practitioners in China) by the end of 2023; our sample represents approximately 0.04% of this national physician population. Provinces with larger sample sizes included Chongqing (894), Henan (239), Heilongjiang (109), Hebei (150), and Hunan (144), while some provinces (such as Qinghai, Hainan, Ningxia, and Jilin) had relatively fewer respondents (Fig. 1A). Additionally, the sample from developed eastern and central regions—such as Shanghai, Beijing, Guangdong, and Sichuan—was adequate.

**Fig. 2:**
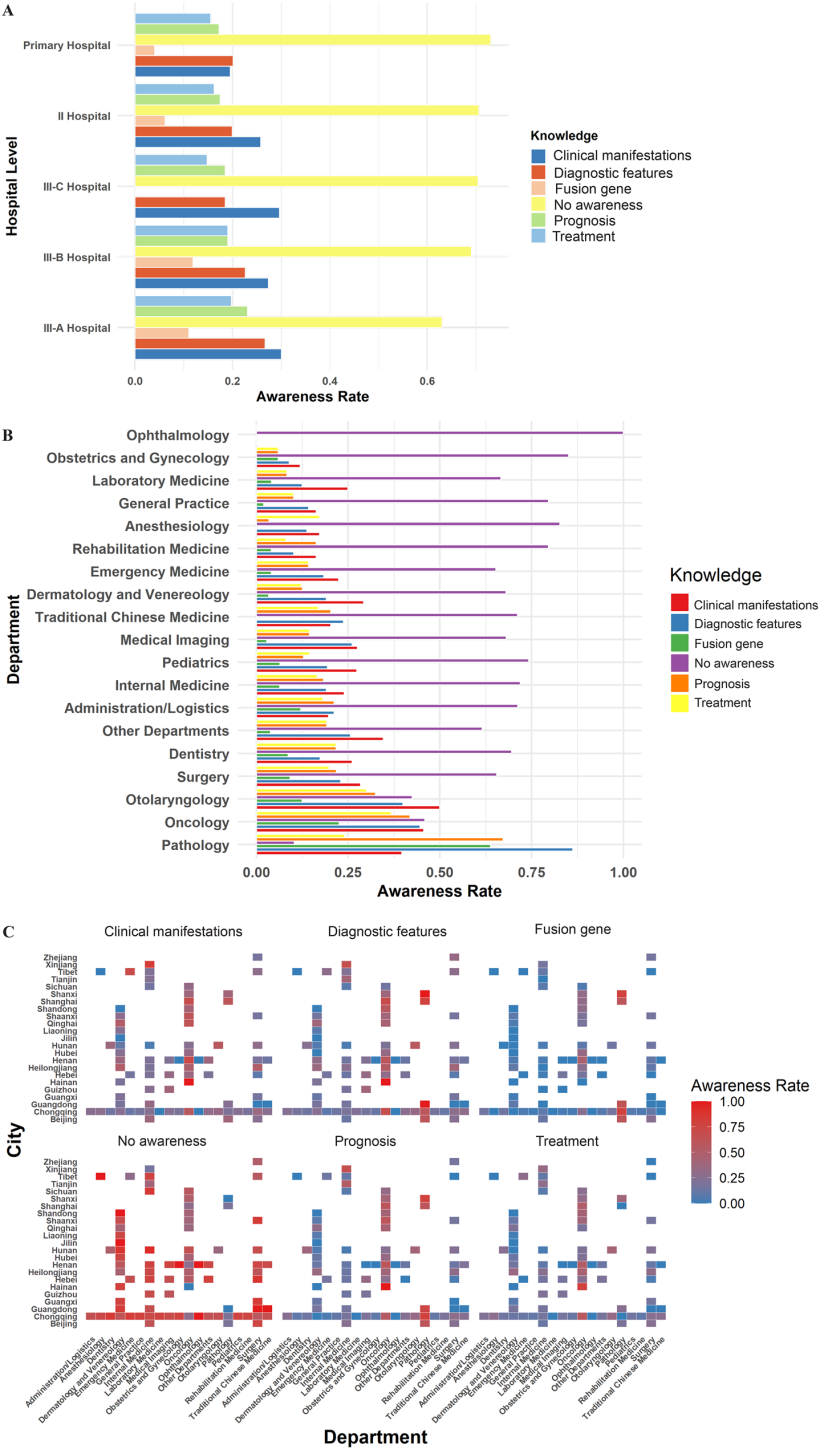
Awareness of NUT carcinoma knowledge stratified by hospital level, department, and city. (**A**) Awareness rates for different knowledge domains (clinical manifestations, diagnostic features, fusion gene, prognosis, treatment, and no knowledge) among clinicians, stratified by hospital level (primary, secondary, and tertiary hospitals). (**B**) Department-specific awareness rates for each knowledge domain. The analysis covers a wide array of clinical and technical specialties, highlighting disparities between related (e.g., oncology, pathology) and unrelated departments. (**C**) Heatmap of awareness rates for each knowledge domain, stratified by both city and department. The intensity of color reflects the proportion of respondents in each city–department pair with knowledge of the corresponding aspect. Notable regional and specialty-related disparities are apparent, with higher awareness in oncology, pathology, and otolaryngology, and lower awareness in non-related departments and central-western provinces

A majority of respondents (81.2%) were from tertiary hospitals, followed by secondary hospitals and a small proportion from primary care institutions (Fig. 2A). In terms of departmental distribution (Fig. 2B), participants were drawn from various relevant clinical and technical departments, including respiratory medicine, oncology, thoracic surgery, pediatrics, and pathology, with respiratory medicine and oncology being predominant.

Nationwide, overall awareness of NUT carcinoma among healthcare professionals was low. Specifically, 28.5% were aware of the clinical manifestations, 24.9% of diagnostic features, 21.6% of prognosis, 18.8% of treatment, and only 9.7% knew about the NUT carcinoma fusion gene. Notably, 65.2% of respondents reported no knowledge of any of these aspects. Awareness of relevant professional organizations was similarly limited (Fig. 1C): only 29.98% knew of the “NUT Carcinoma Gene Diagnosis Working Group, Chinese Anti-Cancer Association,” while awareness of the “NUT Carcinoma Registry” and the “European Cooperative Study Group for Pediatric Rare Tumors” was 10.1% and 9.7%, respectively. Awareness rates of organizations related to NUT carcinoma and different knowledge aspects among survey respondents are detailed in Supplementary Table 1–2, respectively. A total of 68.8% of respondents were unaware of any related organizations.

Significant differences in awareness were observed across regions, departments, and cities. Heatmap analysis further revealed pronounced disparities in the dissemination of NUT carcinoma knowledge among cities and departments nationwide (Fig. 2C). Awareness rates by city and department are shown in Supplementary Table 3–4. In certain provinces—such as Xinjiang (77.8%), Hainan (55.6%), and Shanghai (45.2%)—specialties like oncology, pathology, and otolaryngology had clinical manifestation and diagnostic knowledge rates exceeding 0.75. In contrast, most non-related departments (e.g., ophthalmology, obstetrics and gynecology, anesthesiology, general practice) and central-western regions (e.g., Jilin, Inner Mongolia, Guangxi, Hunan) had awareness rates below 0.25, with the “Know None” proportion often exceeding 0.75 and reaching over 70% in some areas (Fig. 1B). Knowledge of the fusion gene was the lowest, with over 80% of cities and departments reporting awareness rates below 0.25. Awareness of prognosis and treatment was generally poor, with notable improvement only in certain departments in select provinces (e.g., “Awareness of prognosis” in pathology at 67.2%, “Awareness of fusion gene” at 63.8%). Departmentally, oncology, otolaryngology, and pathology had significantly higher rates of clinical manifestation awareness (45.6%, 50.0%, and 39.7%, respectively), while non-related departments had “Know None” rates exceeding 80%. These findings highlight the urgent need to strengthen the dissemination of NUT carcinoma knowledge nationwide, particularly in central and southwestern regions, among non-related specialties, and regarding molecular aspects such as fusion genes, to promote more equitable and advanced clinical practice.

Univariate analysis showed that oncology and pathology professionals had significantly higher awareness of NUTM1 fusion partners compared to other departments (Bonferroni-corrected *p* < 0.001). Detailed *p*-values and statistical results are presented in Supplementary Table 5. To control for confounding and address multicollinearity, multivariable ridge regression (glmnet_ridge) was further employed. All models used five-fold cross-validation to select the optimal penalty parameter (lambda), with bootstrap resampling (*R* = 200) for robust estimation of coefficients and confidence intervals. Significant factors identified by multivariable ridge regression are presented in Supplementary Table 5 and Fig. 1D. Significant results (adjustedα = 9.26 × 10^− 5^) indicated that oncology and pathology professionals had markedly higher awareness across all knowledge domains. For clinical knowledge (OR = 1.66), diagnostic knowledge (oncology OR = 2.04, pathology OR = 9.38), and fusion gene knowledge (OR = 6.49), rates were especially prominent in these departments (*p* < 0.001). In contrast, ophthalmology and anesthesiology had significantly lower awareness, particularly for diagnostic and treatment knowledge, with ophthalmology showing the lowest rates (OR = 0.27–0.35). Regionally, remote provinces such as Inner Mongolia, Gansu, and Yunnan exhibited lower awareness, especially regarding Awareness of prognosis and treatment. These results underscore the pivotal role of oncology and pathology in NUT carcinoma knowledge dissemination and the significant impact of geographic disparities.

The area under the curve (AUC) for all multivariable ridge regression models ranged from 0.65 to 0.79, and Brier scores ranged from 0.07 to 0.20, indicating good model discrimination and predictive accuracy. Several models showed statistically significant Hosmer–Lemeshow test results (*p* < 0.05), suggesting potential miscalibration; therefore, model performance was primarily evaluated using AUC and Brier score, with calibration curves provided in the Supplementary materials (Supplementary Table 6). Notably, certain variables (e.g., some cities) exhibited high variance inflation factors, indicating multicollinearity; however, the ridge regression approach effectively mitigated its impact on model stability. Model performance metrics are summarized in Supplementary Table 6.

## Discussion

This study represents the first nationwide survey to systematically assess Chinese clinicians’ awareness and knowledge of NUT carcinoma. The results reveal that, even among physicians working in oncology-related specialties and tertiary hospitals, the level of understanding regarding NUT carcinoma remains concerningly low. Only 7.0% of respondents demonstrated relatively comprehensive knowledge of NUT carcinoma, while 65.2% reported having never heard of the disease. The knowledge gap is particularly pronounced among healthcare providers in primary medical institutions and non-oncology specialties, and is evident not only in clinical diagnosis and management but also in cutting-edge areas such as molecular pathology. Our survey achieved broad geographic coverage across 31 provinces, autonomous regions, and municipalities in China, with adequate representation from developed eastern and central regions such as Shanghai, Beijing, Guangdong, and Sichuan. The sample included clinicians from various hospital levels, with the majority (81.2%) from tertiary hospitals, followed by secondary hospitals and a small proportion from primary care institutions, reflecting the knowledge status across different tiers of the healthcare system. Participants were drawn from a wide array of clinical and technical departments, including respiratory medicine, oncology, thoracic surgery, pediatrics, and pathology, ensuring coverage of the multidisciplinary teams potentially involved in NUT carcinoma diagnosis and management. Despite this comprehensive sampling framework, awareness remained concerningly low across all levels and specialties, underscoring the pervasive nature of the knowledge gap.

Deficiencies in knowledge are not limited to general departments or junior physicians: even in tertiary hospitals, over one-fifth of clinicians had never encountered a suspected case, and only a minority were aware of relevant professional organizations or guidelines. Awareness of molecular pathology—especially fusion gene partners, which are central to the diagnosis of NUT carcinoma—was extremely low, at less than 10%. This highlights the need for education not only on clinical features and recognition but also on advances in molecular diagnostics. Although oncologists and pathologists showed significantly higher awareness of NUT carcinoma, particularly regarding fusion genes, the overall level of knowledge remains suboptimal. Geographically, healthcare professionals in eastern coastal and major metropolitan areas exhibited higher awareness compared to those in central and western regions, likely reflecting disparities in access to continuing medical education, case exposure, and institutional resources.

NUT carcinoma is a rare and highly aggressive malignancy that is often misdiagnosed as other undifferentiated carcinomas or sarcomas. Early recognition is critical for timely diagnosis and treatment, yet most Chinese physicians do not consider NUT carcinoma in their differential diagnosis, further increasing the risk of misdiagnosis.

Previous studies have shown that patients with rare diseases commonly face diagnostic delays, misdiagnosis, and limited treatment options, with insufficient clinician awareness being a key underlying cause [7–9]. The low level of awareness among Chinese clinicians is consistent with international surveys on NUT carcinoma and other rare tumors. NUT carcinoma is driven by NUT fusion oncoproteins and, although diagnostic methods such as NUT immunohistochemistry are straightforward and direct, it is not classified within lung or head and neck squamous cell carcinoma (SCC) categories. Consequently, oncologists, surgeons, and pathologists often lack awareness of NUT carcinoma [10]. As a result, suspected cases are rarely tested for NUT protein, exacerbating underdiagnosis and misdiagnosis. In fact, despite an annual incidence of approximately 1400 cases in the United States—exceeding that of some rare non-small cell lung cancer (NSCLC) and head and neck squamous cell carcinoma (HNSCC) subtypes—most physicians still regard NUT carcinoma as extremely rare and seldom consider it diagnostically, causing patients to miss opportunities for early diagnosis and targeted therapy. The median survival for NUT carcinoma patients is only about 6.5 months, making early diagnosis crucial for improving prognosis [5]. However, due to low awareness, most patients are diagnosed at advanced stages and often miss timely enrollment in clinical trials or access to standardized treatment. As demonstrated by Zhang et al. [9], in a survey of Chinese clinicians’ awareness of rare diseases, lack of knowledge is a key factor contributing to delayed and erroneous diagnoses of rare tumors.

Given this incidence estimate and China’s large population, the potential number of NUT carcinoma patients in China is considerable. To improve diagnosis and treatment, China’s first NUT carcinoma specialty center was established in 2023, along with the NUT Carcinoma Gene Diagnosis Working Group under the Chinese Anti-Cancer Association. Since 2022, our team has conducted a real-world cohort study (NCT06073938,Registered on: 2023–10-10) evaluating the efficacy of BET inhibitors(NHWD-870) in NUT-rearranged solid tumors [11–13]. Between June 2023 and June 2025, 170 NUT carcinoma patients have already been diagnosed and treated, but due to under-recognition and misdiagnosis, the actual number of cases may be underestimated. To enhance awareness among the public and healthcare professionals, we have collaborated with international experts to establish the NUT Carcinoma Knowledge Platform (http://resource.yin-lab.com/NUT/), which provides authoritative, up-to-date information and maintains a comprehensive registry of published cases and available cohort data to facilitate research into this rare disease. We acknowledge that current therapeutic options for NUT carcinoma remain limited, with many targeted therapies still under clinical investigation. However, improved awareness can facilitate patient enrollment in clinical trials and encourage exploration of treatment strategies within existing frameworks. When possible, patients should be referred to centers with access to clinical trials and emerging targeted approaches.

Despite these initial advances, improving the diagnosis of rare tumors like NUT carcinoma will require multifaceted strategies centered on pathology services and tumor-related specialties. First, training programs should prioritize pathologists to enhance their ability to recognize NUT carcinoma and appropriately utilize NUT immunohistochemistry and molecular confirmation. Rare tumor content should be integrated into medical education and continuing professional development to address diagnostic delays stemming from insufficient exposure during training [8, 9]. Second, awareness among tumor-related clinical specialties (such as oncology, thoracic surgery, and otolaryngology) should be strengthened to ensure that clinicians consider NUT carcinoma in the differential diagnosis of poorly differentiated tumors and request appropriate testing or referral. Dedicated case discussions and multidisciplinary team (MDT) training can enhance diagnostic sensitivity. Third, referral networks should be established to ensure that suspected cases are directed to centers with access to NUT immunohistochemistry and molecular diagnostics, with the promotion of telemedicine expert consultations, particularly in primary and secondary hospitals [7]. While broad dissemination of detailed molecular knowledge to all clinicians may not be practical or necessary, targeted education for pathology and tumor-related specialties can meaningfully reduce diagnostic delays and improve patient outcomes. As more pathologists and clinicians deepen their understanding of NUT carcinoma, the standard of diagnosis and treatment will gradually improve, ultimately benefiting more patients with this rare malignancy.

This study has several limitations that should be considered when interpreting the results. First, although we included a large number of clinicians from different regions and hospital levels across China, the sample represents only a very small proportion of the total physician population nationwide. Participation was voluntary, which likely introduced selection bias—clinicians with greater interest in rare tumors or NUT carcinoma may have been more likely to participate, potentially leading to an overestimation of overall awareness. Second, the sample was predominantly from tertiary hospitals (81.2%), which limits generalizability to primary and secondary care settings where awareness may be even lower. Additionally, regional sample sizes were uneven, with some provinces having very few respondents, which may affect the reliability of regional comparisons. Third, the survey relied on self-reported knowledge and experience, which may not accurately reflect actual clinical practice or diagnostic behavior. Information bias may exist, such as overestimation of knowledge or underestimation of diagnostic uncertainty. Moreover, the questionnaire did not collect information on respondents’ undergraduate education or continuing medical education history, which could have influenced the results. Fourth, as a cross-sectional study, causality cannot be inferred, and the findings only reflect the knowledge status at a single point in time. With the rapid progress in the diagnosis and treatment of rare tumors, the generalizability of the results may be affected over time. Finally, although the questionnaire was designed and piloted by experts, some items were specifically developed for this study and have not undergone systematic psychometric validation.

In summary, while this nationwide survey provides important evidence for understanding the awareness of NUT carcinoma among Chinese clinicians, the above limitations should be fully considered when interpreting the results.

## Conclusions

This nationwide survey reveals that most clinicians in China reported limited awareness of NUT carcinoma, even in tertiary hospitals and tumor-related departments. While definitive diagnosis of NUT carcinoma relies on pathology and molecular confirmation, awareness among tumor-related clinical specialties is essential to prompt appropriate biopsy, request NUT immunohistochemistry or molecular testing, and facilitate timely referral to equipped centers. Practical strategies should therefore prioritize training for pathology services and tumor-related specialties, strengthen referral pathways, and improve access to NUT immunohistochemistry and molecular diagnostics to reduce missed or delayed diagnoses.

## Electronic supplementary material

Below is the link to the electronic supplementary material.

Supplementary material 1

## Data Availability

All relevant data are presented in this article or included in the supplementary materials. The datasets used and analysed during the current study are available from the corresponding author on reasonable request.

## Abbreviations

AIC: Akaike Information Criterion
AUC: Area under the curve
CACA: Chinese Anti-Cancer Association
HNSCC: Head and neck squamous cell carcinoma
LRT: Likelihood ratio test
MDT: Multidisciplinary team
NSCLC: Non-small cell lung cancer
NUT: Nuclear protein in testis
*NUTM1*: Nuclear protein in testis midline carcinoma family member 1 gene
OR: Odds ratio
SCC: Squamous cell carcinoma
VIF: Variance inflation factor
