# Supplementary material for: A nationwide survey of awareness and understanding of NUT carcinoma among clinicians in China
Source: BMC Health Serv Res. 2026 Mar 2;26:477. doi: 10.1186/s12913-026-14236-4 (PMC13059589; doi:10.1186/s12913-026-14236-4)
Supplement: Supplementary file 1 — Supplementary material 1 [file 12913_2026_14236_MOESM1_ESM.docx]

### ****Supplementary Materials****

[Additional fle 1. List of questions. 2](#_Toc13147)

[Supplementary Table 1. Awareness Rates of Organizations Related to NUT Carcinoma Among Survey Respondents 3](#_Toc22707)

[Supplementary Table 2. Awareness Rates of Different Aspects of NUT Carcinoma Among Survey Respondents 3](#_Toc30225)

[Supplementary Table 3. Awareness Rates of Different Aspects of NUT Carcinoma by City 4](#_Toc14900)

[Supplementary Table 4. Awareness Rates of Different Aspects of NUT Carcinoma by City and Department 9](#_Toc3014)

[Supplementary Table 5. Significant Factors Associated with Awareness of NUT Carcinoma Knowledge: Results from Multivariable Ridge Regression Analysise 30](#_Toc9491)

[Supplementary Table 6. Model Performance Metrics of Multivariable Ridge Regression for Each Outcome Variable 38](#_Toc14158)

[Supplementary Methods. Statistical analysis 39](#_Toc14158)

# Additional fle 1. List of questions.

**Questionnaire (English Version)**

1. City where you work:

2. Department where you work:

3. Hospital level where you work:

Tertiary Grade A

Tertiary Grade B

Tertiary Grade C

Secondary Grade A / Secondary Grade B / Secondary Grade C

Primary hospital (community hospital/health center)

4. Which of the following aspects of NUT carcinoma are you relatively familiar with?

(Multiple choices)

Main clinical manifestations of NUT carcinoma patients

Main diagnostic methods for NUT carcinoma

Main treatment methods for NUT carcinoma

Prognosis of NUT carcinoma patients

Common molecular fusion partners of NUT carcinoma

Not familiar with any of the above

5. Have you heard of any of the following organizations or groups related to NUT carcinoma?

(Multiple choices)

A. NUT Carcinoma Genetic Diagnosis Working Group, Cancer Genetics Diagnosis Committee, Chinese Anti-Cancer Association

B. NUT Carcinoma Registry

C. European Cooperative Study Group for Rare Pediatric Tumors

1. None of the above

# Supplementary Table 1. Awareness Rates of Organizations Related to NUT Carcinoma Among Survey Respondents

| **Organization** | **AwarenessRate** |
| --- | --- |
| NUT Carcinoma Gene Diagnosis Working Group, Chinese Anti-Cancer Association | 0.299774266365688 |
| NUT Carcinoma Registry | 0.101128668171558 |
| European Cooperative Study Group for Pediatric Rare Tumors | 0.0966139954853273 |
| None known | 0.687584650112867 |

Awareness rate refers to the proportion of respondents who reported being aware of each respective organization or group related to NUT carcinoma. "None known" indicates respondents who were not aware of any of the listed organizations.

# Supplementary Table 2. Awareness Rates of Different Aspects of NUT Carcinoma Among Survey Respondents

| **Knowledge** | **AwarenessRate** |
| --- | --- |
| Know Clinical | 0.284875846501129 |
| Know Diagnosis | 0.24920993227991 |
| Know Treatment | 0.188261851015801 |
| Know Prognosis | 0.215801354401806 |
| Know Fusion | 0.0966139954853273 |
| Know None | 0.652370203160271 |

Awareness rate refers to the proportion of respondents who reported being familiar with each specific aspect of NUT carcinoma. "Know None" indicates respondents who were not familiar with any of the listed aspects.

# Supplementary Table 3. Awareness Rates of Different Aspects of NUT Carcinoma by City

| **City** | **AwarenessRate** | **n** | **Knowledge** |
| --- | --- | --- | --- |
| Anhui | 0.2 | 5 | Know Clinical |
| Beijing | 0.317073170731707 | 41 | Know Clinical |
| Chongqing | 0.266219239373602 | 894 | Know Clinical |
| Foreign | 0.5 | 2 | Know Clinical |
| Fujian | 0.333333333333333 | 6 | Know Clinical |
| Gansu | 0.666666666666667 | 3 | Know Clinical |
| Guangdong | 0.183673469387755 | 49 | Know Clinical |
| Guangxi | 0.214285714285714 | 70 | Know Clinical |
| Guizhou | 0.295454545454545 | 44 | Know Clinical |
| Hainan | 0.555555555555556 | 18 | Know Clinical |
| Hebei | 0.24 | 150 | Know Clinical |
| Heilongjiang | 0.440366972477064 | 109 | Know Clinical |
| Henan | 0.267782426778243 | 239 | Know Clinical |
| Hubei | 0.230769230769231 | 26 | Know Clinical |
| Hunan | 0.229166666666667 | 144 | Know Clinical |
| Inner Mongolia | 0.142857142857143 | 7 | Know Clinical |
| Jiangsu | 0.25 | 16 | Know Clinical |
| Jiangxi | 0.625 | 8 | Know Clinical |
| Jilin | 0.2 | 10 | Know Clinical |
| Liaoning | 0.234042553191489 | 47 | Know Clinical |
| Ningxia | 0.166666666666667 | 6 | Know Clinical |
| Qinghai | 0.513513513513513 | 37 | Know Clinical |
| Shaanxi | 0.307692307692308 | 39 | Know Clinical |
| Shandong | 0.3125 | 16 | Know Clinical |
| Shanghai | 0.451612903225806 | 31 | Know Clinical |
| Shanxi | 0.382978723404255 | 47 | Know Clinical |
| Sichuan | 0.296296296296296 | 54 | Know Clinical |
| Tianjin | 0.241379310344828 | 29 | Know Clinical |
| Tibet | 0.290322580645161 | 31 | Know Clinical |
| Xinjiang | 0.777777777777778 | 9 | Know Clinical |
| Yunnan | 0.333333333333333 | 3 | Know Clinical |
| Zhejiang | 0.32 | 25 | Know Clinical |
| Anhui | 0.4 | 5 | Know Diagnosis |
| Beijing | 0.268292682926829 | 41 | Know Diagnosis |
| Chongqing | 0.227069351230425 | 894 | Know Diagnosis |
| Foreign | 0.5 | 2 | Know Diagnosis |
| Fujian | 0.166666666666667 | 6 | Know Diagnosis |
| Gansu | 0.333333333333333 | 3 | Know Diagnosis |
| Guangdong | 0.285714285714286 | 49 | Know Diagnosis |
| Guangxi | 0.142857142857143 | 70 | Know Diagnosis |
| Guizhou | 0.227272727272727 | 44 | Know Diagnosis |
| Hainan | 0.555555555555556 | 18 | Know Diagnosis |
| Hebei | 0.213333333333333 | 150 | Know Diagnosis |
| Heilongjiang | 0.431192660550459 | 109 | Know Diagnosis |
| Henan | 0.209205020920502 | 239 | Know Diagnosis |
| Hubei | 0.230769230769231 | 26 | Know Diagnosis |
| Hunan | 0.222222222222222 | 144 | Know Diagnosis |
| Inner Mongolia | 0.142857142857143 | 7 | Know Diagnosis |
| Jiangsu | 0.25 | 16 | Know Diagnosis |
| Jiangxi | 0.25 | 8 | Know Diagnosis |
| Jilin | 0.1 | 10 | Know Diagnosis |
| Liaoning | 0.0638297872340425 | 47 | Know Diagnosis |
| Ningxia | 0.333333333333333 | 6 | Know Diagnosis |
| Qinghai | 0.378378378378378 | 37 | Know Diagnosis |
| Shaanxi | 0.230769230769231 | 39 | Know Diagnosis |
| Shandong | 0.4375 | 16 | Know Diagnosis |
| Shanghai | 0.419354838709677 | 31 | Know Diagnosis |
| Shanxi | 0.446808510638298 | 47 | Know Diagnosis |
| Sichuan | 0.277777777777778 | 54 | Know Diagnosis |
| Tianjin | 0.275862068965517 | 29 | Know Diagnosis |
| Tibet | 0.193548387096774 | 31 | Know Diagnosis |
| Xinjiang | 0.777777777777778 | 9 | Know Diagnosis |
| Yunnan | 0.666666666666667 | 3 | Know Diagnosis |
| Zhejiang | 0.28 | 25 | Know Diagnosis |
| Anhui | 0.2 | 5 | Know Treatment |
| Beijing | 0.195121951219512 | 41 | Know Treatment |
| Chongqing | 0.194630872483221 | 894 | Know Treatment |
| Foreign | 0.5 | 2 | Know Treatment |
| Fujian | 0.166666666666667 | 6 | Know Treatment |
| Gansu | 0 | 3 | Know Treatment |
| Guangdong | 0.142857142857143 | 49 | Know Treatment |
| Guangxi | 0.114285714285714 | 70 | Know Treatment |
| Guizhou | 0.181818181818182 | 44 | Know Treatment |
| Hainan | 0.388888888888889 | 18 | Know Treatment |
| Hebei | 0.16 | 150 | Know Treatment |
| Heilongjiang | 0.357798165137615 | 109 | Know Treatment |
| Henan | 0.125523012552301 | 239 | Know Treatment |
| Hubei | 0.153846153846154 | 26 | Know Treatment |
| Hunan | 0.166666666666667 | 144 | Know Treatment |
| Inner Mongolia | 0.142857142857143 | 7 | Know Treatment |
| Jiangsu | 0.25 | 16 | Know Treatment |
| Jiangxi | 0 | 8 | Know Treatment |
| Jilin | 0 | 10 | Know Treatment |
| Liaoning | 0.0212765957446809 | 47 | Know Treatment |
| Ningxia | 0.166666666666667 | 6 | Know Treatment |
| Qinghai | 0.189189189189189 | 37 | Know Treatment |
| Shaanxi | 0.179487179487179 | 39 | Know Treatment |
| Shandong | 0.3125 | 16 | Know Treatment |
| Shanghai | 0.258064516129032 | 31 | Know Treatment |
| Shanxi | 0.319148936170213 | 47 | Know Treatment |
| Sichuan | 0.277777777777778 | 54 | Know Treatment |
| Tianjin | 0.206896551724138 | 29 | Know Treatment |
| Tibet | 0.0967741935483871 | 31 | Know Treatment |
| Xinjiang | 0.333333333333333 | 9 | Know Treatment |
| Yunnan | 0.333333333333333 | 3 | Know Treatment |
| Zhejiang | 0.16 | 25 | Know Treatment |
| Anhui | 0.2 | 5 | Know Prognosis |
| Beijing | 0.292682926829268 | 41 | Know Prognosis |
| Chongqing | 0.211409395973154 | 894 | Know Prognosis |
| Foreign | 0.5 | 2 | Know Prognosis |
| Fujian | 0.333333333333333 | 6 | Know Prognosis |
| Gansu | 0.666666666666667 | 3 | Know Prognosis |
| Guangdong | 0.244897959183673 | 49 | Know Prognosis |
| Guangxi | 0.114285714285714 | 70 | Know Prognosis |
| Guizhou | 0.136363636363636 | 44 | Know Prognosis |
| Hainan | 0.388888888888889 | 18 | Know Prognosis |
| Hebei | 0.173333333333333 | 150 | Know Prognosis |
| Heilongjiang | 0.302752293577982 | 109 | Know Prognosis |
| Henan | 0.146443514644351 | 239 | Know Prognosis |
| Hubei | 0.192307692307692 | 26 | Know Prognosis |
| Hunan | 0.194444444444444 | 144 | Know Prognosis |
| Inner Mongolia | 0 | 7 | Know Prognosis |
| Jiangsu | 0.375 | 16 | Know Prognosis |
| Jiangxi | 0.25 | 8 | Know Prognosis |
| Jilin | 0.2 | 10 | Know Prognosis |
| Liaoning | 0.0638297872340425 | 47 | Know Prognosis |
| Ningxia | 0.5 | 6 | Know Prognosis |
| Qinghai | 0.324324324324324 | 37 | Know Prognosis |
| Shaanxi | 0.205128205128205 | 39 | Know Prognosis |
| Shandong | 0.375 | 16 | Know Prognosis |
| Shanghai | 0.419354838709677 | 31 | Know Prognosis |
| Shanxi | 0.425531914893617 | 47 | Know Prognosis |
| Sichuan | 0.240740740740741 | 54 | Know Prognosis |
| Tianjin | 0.344827586206897 | 29 | Know Prognosis |
| Tibet | 0.0645161290322581 | 31 | Know Prognosis |
| Xinjiang | 0.444444444444444 | 9 | Know Prognosis |
| Yunnan | 0.333333333333333 | 3 | Know Prognosis |
| Zhejiang | 0.24 | 25 | Know Prognosis |
| Anhui | 0.2 | 5 | Know Fusion |
| Beijing | 0.268292682926829 | 41 | Know Fusion |
| Chongqing | 0.0850111856823266 | 894 | Know Fusion |
| Foreign | 0.5 | 2 | Know Fusion |
| Fujian | 0.166666666666667 | 6 | Know Fusion |
| Gansu | 0.333333333333333 | 3 | Know Fusion |
| Guangdong | 0.122448979591837 | 49 | Know Fusion |
| Guangxi | 0.0428571428571429 | 70 | Know Fusion |
| Guizhou | 0.0227272727272727 | 44 | Know Fusion |
| Hainan | 0 | 18 | Know Fusion |
| Hebei | 0.08 | 150 | Know Fusion |
| Heilongjiang | 0.165137614678899 | 109 | Know Fusion |
| Henan | 0.0753138075313808 | 239 | Know Fusion |
| Hubei | 0.153846153846154 | 26 | Know Fusion |
| Hunan | 0.0833333333333333 | 144 | Know Fusion |
| Inner Mongolia | 0 | 7 | Know Fusion |
| Jiangsu | 0.1875 | 16 | Know Fusion |
| Jiangxi | 0 | 8 | Know Fusion |
| Jilin | 0.1 | 10 | Know Fusion |
| Liaoning | 0 | 47 | Know Fusion |
| Ningxia | 0 | 6 | Know Fusion |
| Qinghai | 0.0810810810810811 | 37 | Know Fusion |
| Shaanxi | 0.128205128205128 | 39 | Know Fusion |
| Shandong | 0.3125 | 16 | Know Fusion |
| Shanghai | 0.225806451612903 | 31 | Know Fusion |
| Shanxi | 0.319148936170213 | 47 | Know Fusion |
| Sichuan | 0.0555555555555556 | 54 | Know Fusion |
| Tianjin | 0 | 29 | Know Fusion |
| Tibet | 0.032258064516129 | 31 | Know Fusion |
| Xinjiang | 0.111111111111111 | 9 | Know Fusion |
| Yunnan | 0.333333333333333 | 3 | Know Fusion |
| Zhejiang | 0.16 | 25 | Know Fusion |
| Anhui | 0.6 | 5 | Know None |
| Beijing | 0.585365853658537 | 41 | Know None |
| Chongqing | 0.678970917225951 | 894 | Know None |
| Foreign | 1 | 2 | Know None |
| Fujian | 0.666666666666667 | 6 | Know None |
| Gansu | 0 | 3 | Know None |
| Guangdong | 0.653061224489796 | 49 | Know None |
| Guangxi | 0.771428571428571 | 70 | Know None |
| Guizhou | 0.659090909090909 | 44 | Know None |
| Hainan | 0.444444444444444 | 18 | Know None |
| Hebei | 0.693333333333333 | 150 | Know None |
| Heilongjiang | 0.541284403669725 | 109 | Know None |
| Henan | 0.694560669456067 | 239 | Know None |
| Hubei | 0.615384615384615 | 26 | Know None |
| Hunan | 0.701388888888889 | 144 | Know None |
| Inner Mongolia | 0.857142857142857 | 7 | Know None |
| Jiangsu | 0.5 | 16 | Know None |
| Jiangxi | 0.25 | 8 | Know None |
| Jilin | 0.8 | 10 | Know None |
| Liaoning | 0.74468085106383 | 47 | Know None |
| Ningxia | 0.5 | 6 | Know None |
| Qinghai | 0.459459459459459 | 37 | Know None |
| Shaanxi | 0.641025641025641 | 39 | Know None |
| Shandong | 0.5625 | 16 | Know None |
| Shanghai | 0.451612903225806 | 31 | Know None |
| Shanxi | 0.468085106382979 | 47 | Know None |
| Sichuan | 0.611111111111111 | 54 | Know None |
| Tianjin | 0.551724137931034 | 29 | Know None |
| Tibet | 0.67741935483871 | 31 | Know None |
| Xinjiang | 0.111111111111111 | 9 | Know None |
| Yunnan | 0.333333333333333 | 3 | Know None |
| Zhejiang | 0.6 | 25 | Know None |

Awareness rate refers to the proportion of respondents from each city who reported being familiar with a specific aspect of NUT carcinoma. “n” represents the number of respondents from each city for the corresponding knowledge aspect. “Knowledge” indicates the specific aspect of NUT carcinoma (clinical manifestations, diagnosis, treatment, prognosis, molecular fusion partners, or none) that the awareness rate refers to.

# Supplementary Table 4. Awareness Rates of Different Aspects of NUT Carcinoma by City and Department

| **City** | **Department** | **AwarenessRate** | **n** | **Knowledge** |
| --- | --- | --- | --- | --- |
| Beijing | Internal Medicine | 0.375 | 8 | Know Clinical |
| Beijing | Pathology | 0.4 | 5 | Know Clinical |
| Beijing | Surgery | 0.25 | 8 | Know Clinical |
| Chongqing | Administration/Logistics | 0.254901960784314 | 51 | Know Clinical |
| Chongqing | Anesthesiology | 0.272727272727273 | 11 | Know Clinical |
| Chongqing | Dentistry | 0.142857142857143 | 7 | Know Clinical |
| Chongqing | Dermatology and Venereology | 0.25 | 8 | Know Clinical |
| Chongqing | Emergency Medicine | 0.125 | 24 | Know Clinical |
| Chongqing | General Practice | 0.170212765957447 | 47 | Know Clinical |
| Chongqing | Internal Medicine | 0.244755244755245 | 286 | Know Clinical |
| Chongqing | Laboratory Medicine | 0 | 9 | Know Clinical |
| Chongqing | Medical Imaging | 0.233333333333333 | 30 | Know Clinical |
| Chongqing | Obstetrics and Gynecology | 0.1875 | 32 | Know Clinical |
| Chongqing | Oncology | 0.571428571428571 | 35 | Know Clinical |
| Chongqing | Ophthalmology | 0 | 8 | Know Clinical |
| Chongqing | Other Departments | 0.288888888888889 | 45 | Know Clinical |
| Chongqing | Otolaryngology | 0.333333333333333 | 6 | Know Clinical |
| Chongqing | Pathology | 0.2 | 5 | Know Clinical |
| Chongqing | Pediatrics | 0.244444444444444 | 45 | Know Clinical |
| Chongqing | Rehabilitation Medicine | 0.195121951219512 | 41 | Know Clinical |
| Chongqing | Surgery | 0.358381502890173 | 173 | Know Clinical |
| Chongqing | Traditional Chinese Medicine | 0.258064516129032 | 31 | Know Clinical |
| Guangdong | Dermatology and Venereology | 0.181818181818182 | 11 | Know Clinical |
| Guangdong | Internal Medicine | 0.333333333333333 | 6 | Know Clinical |
| Guangdong | Pathology | 0.25 | 8 | Know Clinical |
| Guangdong | Surgery | 0 | 5 | Know Clinical |
| Guangdong | Traditional Chinese Medicine | 0 | 6 | Know Clinical |
| Guangxi | Dermatology and Venereology | 0.214285714285714 | 42 | Know Clinical |
| Guangxi | Surgery | 0.166666666666667 | 6 | Know Clinical |
| Guizhou | Internal Medicine | 0.25 | 28 | Know Clinical |
| Guizhou | Medical Imaging | 0.4 | 10 | Know Clinical |
| Hainan | Dermatology and Venereology | 0.2 | 5 | Know Clinical |
| Hainan | Oncology | 1 | 5 | Know Clinical |
| Hebei | Emergency Medicine | 0.166666666666667 | 6 | Know Clinical |
| Hebei | Internal Medicine | 0.204545454545455 | 44 | Know Clinical |
| Hebei | Medical Imaging | 0.333333333333333 | 6 | Know Clinical |
| Hebei | Oncology | 0.347826086956522 | 46 | Know Clinical |
| Hebei | Other Departments | 0.2 | 5 | Know Clinical |
| Hebei | Surgery | 0.08 | 25 | Know Clinical |
| Heilongjiang | Dermatology and Venereology | 0.5 | 20 | Know Clinical |
| Heilongjiang | Internal Medicine | 0.2 | 15 | Know Clinical |
| Heilongjiang | Oncology | 0.490566037735849 | 53 | Know Clinical |
| Heilongjiang | Surgery | 0.5 | 10 | Know Clinical |
| Henan | Dermatology and Venereology | 0.370967741935484 | 62 | Know Clinical |
| Henan | Internal Medicine | 0.207547169811321 | 53 | Know Clinical |
| Henan | Medical Imaging | 0.222222222222222 | 9 | Know Clinical |
| Henan | Obstetrics and Gynecology | 0 | 12 | Know Clinical |
| Henan | Oncology | 0.733333333333333 | 15 | Know Clinical |
| Henan | Ophthalmology | 0 | 7 | Know Clinical |
| Henan | Other Departments | 0.375 | 8 | Know Clinical |
| Henan | Surgery | 0.194444444444444 | 36 | Know Clinical |
| Henan | Traditional Chinese Medicine | 0.142857142857143 | 14 | Know Clinical |
| Hubei | Dermatology and Venereology | 0.25 | 8 | Know Clinical |
| Hubei | Oncology | 0.333333333333333 | 9 | Know Clinical |
| Hunan | Dentistry | 0.375 | 8 | Know Clinical |
| Hunan | Dermatology and Venereology | 0.111111111111111 | 18 | Know Clinical |
| Hunan | Internal Medicine | 0.0384615384615385 | 26 | Know Clinical |
| Hunan | Oncology | 0.25 | 24 | Know Clinical |
| Hunan | Otolaryngology | 0.571428571428571 | 14 | Know Clinical |
| Hunan | Surgery | 0.294117647058824 | 34 | Know Clinical |
| Jilin | Dermatology and Venereology | 0 | 8 | Know Clinical |
| Liaoning | Dermatology and Venereology | 0.238095238095238 | 42 | Know Clinical |
| Qinghai | Dermatology and Venereology | 0.521739130434783 | 23 | Know Clinical |
| Qinghai | Oncology | 0.636363636363636 | 11 | Know Clinical |
| Shaanxi | Dermatology and Venereology | 0.28 | 25 | Know Clinical |
| Shaanxi | Oncology | 0.6 | 5 | Know Clinical |
| Shaanxi | Surgery | 0.166666666666667 | 6 | Know Clinical |
| Shandong | Dermatology and Venereology | 0 | 5 | Know Clinical |
| Shandong | Oncology | 0.428571428571429 | 7 | Know Clinical |
| Shanghai | Oncology | 0.7 | 10 | Know Clinical |
| Shanghai | Pathology | 0.6 | 5 | Know Clinical |
| Shanxi | Oncology | 0.388888888888889 | 36 | Know Clinical |
| Shanxi | Pathology | 0.4 | 5 | Know Clinical |
| Sichuan | Internal Medicine | 0.153846153846154 | 13 | Know Clinical |
| Sichuan | Oncology | 0.315789473684211 | 19 | Know Clinical |
| Tianjin | Internal Medicine | 0.235294117647059 | 17 | Know Clinical |
| Tibet | Anesthesiology | 0 | 5 | Know Clinical |
| Tibet | Emergency Medicine | 0.714285714285714 | 7 | Know Clinical |
| Tibet | Internal Medicine | 0.222222222222222 | 9 | Know Clinical |
| Tibet | Surgery | 0.285714285714286 | 7 | Know Clinical |
| Xinjiang | Internal Medicine | 0.833333333333333 | 6 | Know Clinical |
| Zhejiang | Surgery | 0.166666666666667 | 6 | Know Clinical |
| Beijing | Internal Medicine | 0.125 | 8 | Know Diagnosis |
| Beijing | Pathology | 0.6 | 5 | Know Diagnosis |
| Beijing | Surgery | 0.25 | 8 | Know Diagnosis |
| Chongqing | Administration/Logistics | 0.235294117647059 | 51 | Know Diagnosis |
| Chongqing | Anesthesiology | 0.181818181818182 | 11 | Know Diagnosis |
| Chongqing | Dentistry | 0.142857142857143 | 7 | Know Diagnosis |
| Chongqing | Dermatology and Venereology | 0.25 | 8 | Know Diagnosis |
| Chongqing | Emergency Medicine | 0.0833333333333333 | 24 | Know Diagnosis |
| Chongqing | General Practice | 0.148936170212766 | 47 | Know Diagnosis |
| Chongqing | Internal Medicine | 0.192307692307692 | 286 | Know Diagnosis |
| Chongqing | Laboratory Medicine | 0.222222222222222 | 9 | Know Diagnosis |
| Chongqing | Medical Imaging | 0.166666666666667 | 30 | Know Diagnosis |
| Chongqing | Obstetrics and Gynecology | 0.125 | 32 | Know Diagnosis |
| Chongqing | Oncology | 0.685714285714286 | 35 | Know Diagnosis |
| Chongqing | Ophthalmology | 0 | 8 | Know Diagnosis |
| Chongqing | Other Departments | 0.266666666666667 | 45 | Know Diagnosis |
| Chongqing | Otolaryngology | 0.333333333333333 | 6 | Know Diagnosis |
| Chongqing | Pathology | 0.6 | 5 | Know Diagnosis |
| Chongqing | Pediatrics | 0.177777777777778 | 45 | Know Diagnosis |
| Chongqing | Rehabilitation Medicine | 0.121951219512195 | 41 | Know Diagnosis |
| Chongqing | Surgery | 0.277456647398844 | 173 | Know Diagnosis |
| Chongqing | Traditional Chinese Medicine | 0.290322580645161 | 31 | Know Diagnosis |
| Guangdong | Dermatology and Venereology | 0.181818181818182 | 11 | Know Diagnosis |
| Guangdong | Internal Medicine | 0.166666666666667 | 6 | Know Diagnosis |
| Guangdong | Pathology | 1 | 8 | Know Diagnosis |
| Guangdong | Surgery | 0 | 5 | Know Diagnosis |
| Guangdong | Traditional Chinese Medicine | 0 | 6 | Know Diagnosis |
| Guangxi | Dermatology and Venereology | 0.119047619047619 | 42 | Know Diagnosis |
| Guangxi | Surgery | 0.166666666666667 | 6 | Know Diagnosis |
| Guizhou | Internal Medicine | 0.178571428571429 | 28 | Know Diagnosis |
| Guizhou | Medical Imaging | 0.4 | 10 | Know Diagnosis |
| Hainan | Dermatology and Venereology | 0.2 | 5 | Know Diagnosis |
| Hainan | Oncology | 1 | 5 | Know Diagnosis |
| Hebei | Emergency Medicine | 0.166666666666667 | 6 | Know Diagnosis |
| Hebei | Internal Medicine | 0.136363636363636 | 44 | Know Diagnosis |
| Hebei | Medical Imaging | 0.333333333333333 | 6 | Know Diagnosis |
| Hebei | Oncology | 0.304347826086957 | 46 | Know Diagnosis |
| Hebei | Other Departments | 0.2 | 5 | Know Diagnosis |
| Hebei | Surgery | 0.08 | 25 | Know Diagnosis |
| Heilongjiang | Dermatology and Venereology | 0.35 | 20 | Know Diagnosis |
| Heilongjiang | Internal Medicine | 0.266666666666667 | 15 | Know Diagnosis |
| Heilongjiang | Oncology | 0.528301886792453 | 53 | Know Diagnosis |
| Heilongjiang | Surgery | 0.4 | 10 | Know Diagnosis |
| Henan | Dermatology and Venereology | 0.290322580645161 | 62 | Know Diagnosis |
| Henan | Internal Medicine | 0.150943396226415 | 53 | Know Diagnosis |
| Henan | Medical Imaging | 0.222222222222222 | 9 | Know Diagnosis |
| Henan | Obstetrics and Gynecology | 0 | 12 | Know Diagnosis |
| Henan | Oncology | 0.6 | 15 | Know Diagnosis |
| Henan | Ophthalmology | 0 | 7 | Know Diagnosis |
| Henan | Other Departments | 0.125 | 8 | Know Diagnosis |
| Henan | Surgery | 0.166666666666667 | 36 | Know Diagnosis |
| Henan | Traditional Chinese Medicine | 0.142857142857143 | 14 | Know Diagnosis |
| Hubei | Dermatology and Venereology | 0 | 8 | Know Diagnosis |
| Hubei | Oncology | 0.333333333333333 | 9 | Know Diagnosis |
| Hunan | Dentistry | 0.25 | 8 | Know Diagnosis |
| Hunan | Dermatology and Venereology | 0.0555555555555556 | 18 | Know Diagnosis |
| Hunan | Internal Medicine | 0.0384615384615385 | 26 | Know Diagnosis |
| Hunan | Oncology | 0.208333333333333 | 24 | Know Diagnosis |
| Hunan | Otolaryngology | 0.571428571428571 | 14 | Know Diagnosis |
| Hunan | Surgery | 0.235294117647059 | 34 | Know Diagnosis |
| Jilin | Dermatology and Venereology | 0 | 8 | Know Diagnosis |
| Liaoning | Dermatology and Venereology | 0.0714285714285714 | 42 | Know Diagnosis |
| Qinghai | Dermatology and Venereology | 0.391304347826087 | 23 | Know Diagnosis |
| Qinghai | Oncology | 0.454545454545455 | 11 | Know Diagnosis |
| Shaanxi | Dermatology and Venereology | 0.16 | 25 | Know Diagnosis |
| Shaanxi | Oncology | 0.4 | 5 | Know Diagnosis |
| Shaanxi | Surgery | 0.166666666666667 | 6 | Know Diagnosis |
| Shandong | Dermatology and Venereology | 0 | 5 | Know Diagnosis |
| Shandong | Oncology | 0.571428571428571 | 7 | Know Diagnosis |
| Shanghai | Oncology | 0.7 | 10 | Know Diagnosis |
| Shanghai | Pathology | 0.8 | 5 | Know Diagnosis |
| Shanxi | Oncology | 0.388888888888889 | 36 | Know Diagnosis |
| Shanxi | Pathology | 1 | 5 | Know Diagnosis |
| Sichuan | Internal Medicine | 0.0769230769230769 | 13 | Know Diagnosis |
| Sichuan | Oncology | 0.157894736842105 | 19 | Know Diagnosis |
| Tianjin | Internal Medicine | 0.411764705882353 | 17 | Know Diagnosis |
| Tibet | Anesthesiology | 0 | 5 | Know Diagnosis |
| Tibet | Emergency Medicine | 0.285714285714286 | 7 | Know Diagnosis |
| Tibet | Internal Medicine | 0.222222222222222 | 9 | Know Diagnosis |
| Tibet | Surgery | 0.285714285714286 | 7 | Know Diagnosis |
| Xinjiang | Internal Medicine | 0.666666666666667 | 6 | Know Diagnosis |
| Zhejiang | Surgery | 0.333333333333333 | 6 | Know Diagnosis |
| Beijing | Internal Medicine | 0.125 | 8 | Know Treatment |
| Beijing | Pathology | 0.2 | 5 | Know Treatment |
| Beijing | Surgery | 0.25 | 8 | Know Treatment |
| Chongqing | Administration/Logistics | 0.215686274509804 | 51 | Know Treatment |
| Chongqing | Anesthesiology | 0.272727272727273 | 11 | Know Treatment |
| Chongqing | Dentistry | 0.142857142857143 | 7 | Know Treatment |
| Chongqing | Dermatology and Venereology | 0.125 | 8 | Know Treatment |
| Chongqing | Emergency Medicine | 0.125 | 24 | Know Treatment |
| Chongqing | General Practice | 0.106382978723404 | 47 | Know Treatment |
| Chongqing | Internal Medicine | 0.167832167832168 | 286 | Know Treatment |
| Chongqing | Laboratory Medicine | 0 | 9 | Know Treatment |
| Chongqing | Medical Imaging | 0.166666666666667 | 30 | Know Treatment |
| Chongqing | Obstetrics and Gynecology | 0.125 | 32 | Know Treatment |
| Chongqing | Oncology | 0.571428571428571 | 35 | Know Treatment |
| Chongqing | Ophthalmology | 0 | 8 | Know Treatment |
| Chongqing | Other Departments | 0.177777777777778 | 45 | Know Treatment |
| Chongqing | Otolaryngology | 0.333333333333333 | 6 | Know Treatment |
| Chongqing | Pathology | 0 | 5 | Know Treatment |
| Chongqing | Pediatrics | 0.155555555555556 | 45 | Know Treatment |
| Chongqing | Rehabilitation Medicine | 0.0975609756097561 | 41 | Know Treatment |
| Chongqing | Surgery | 0.254335260115607 | 173 | Know Treatment |
| Chongqing | Traditional Chinese Medicine | 0.258064516129032 | 31 | Know Treatment |
| Guangdong | Dermatology and Venereology | 0.181818181818182 | 11 | Know Treatment |
| Guangdong | Internal Medicine | 0.166666666666667 | 6 | Know Treatment |
| Guangdong | Pathology | 0.25 | 8 | Know Treatment |
| Guangdong | Surgery | 0 | 5 | Know Treatment |
| Guangdong | Traditional Chinese Medicine | 0 | 6 | Know Treatment |
| Guangxi | Dermatology and Venereology | 0.0714285714285714 | 42 | Know Treatment |
| Guangxi | Surgery | 0.166666666666667 | 6 | Know Treatment |
| Guizhou | Internal Medicine | 0.214285714285714 | 28 | Know Treatment |
| Guizhou | Medical Imaging | 0.2 | 10 | Know Treatment |
| Hainan | Dermatology and Venereology | 0 | 5 | Know Treatment |
| Hainan | Oncology | 0.8 | 5 | Know Treatment |
| Hebei | Emergency Medicine | 0 | 6 | Know Treatment |
| Hebei | Internal Medicine | 0.159090909090909 | 44 | Know Treatment |
| Hebei | Medical Imaging | 0.166666666666667 | 6 | Know Treatment |
| Hebei | Oncology | 0.260869565217391 | 46 | Know Treatment |
| Hebei | Other Departments | 0.2 | 5 | Know Treatment |
| Hebei | Surgery | 0.04 | 25 | Know Treatment |
| Heilongjiang | Dermatology and Venereology | 0.35 | 20 | Know Treatment |
| Heilongjiang | Internal Medicine | 0.266666666666667 | 15 | Know Treatment |
| Heilongjiang | Oncology | 0.452830188679245 | 53 | Know Treatment |
| Heilongjiang | Surgery | 0.3 | 10 | Know Treatment |
| Henan | Dermatology and Venereology | 0.161290322580645 | 62 | Know Treatment |
| Henan | Internal Medicine | 0.0943396226415094 | 53 | Know Treatment |
| Henan | Medical Imaging | 0 | 9 | Know Treatment |
| Henan | Obstetrics and Gynecology | 0 | 12 | Know Treatment |
| Henan | Oncology | 0.533333333333333 | 15 | Know Treatment |
| Henan | Ophthalmology | 0 | 7 | Know Treatment |
| Henan | Other Departments | 0.125 | 8 | Know Treatment |
| Henan | Surgery | 0.111111111111111 | 36 | Know Treatment |
| Henan | Traditional Chinese Medicine | 0 | 14 | Know Treatment |
| Hubei | Dermatology and Venereology | 0.125 | 8 | Know Treatment |
| Hubei | Oncology | 0.222222222222222 | 9 | Know Treatment |
| Hunan | Dentistry | 0.25 | 8 | Know Treatment |
| Hunan | Dermatology and Venereology | 0 | 18 | Know Treatment |
| Hunan | Internal Medicine | 0.0384615384615385 | 26 | Know Treatment |
| Hunan | Oncology | 0.208333333333333 | 24 | Know Treatment |
| Hunan | Otolaryngology | 0.428571428571429 | 14 | Know Treatment |
| Hunan | Surgery | 0.235294117647059 | 34 | Know Treatment |
| Jilin | Dermatology and Venereology | 0 | 8 | Know Treatment |
| Liaoning | Dermatology and Venereology | 0.0238095238095238 | 42 | Know Treatment |
| Qinghai | Dermatology and Venereology | 0.217391304347826 | 23 | Know Treatment |
| Qinghai | Oncology | 0.181818181818182 | 11 | Know Treatment |
| Shaanxi | Dermatology and Venereology | 0.12 | 25 | Know Treatment |
| Shaanxi | Oncology | 0.4 | 5 | Know Treatment |
| Shaanxi | Surgery | 0.166666666666667 | 6 | Know Treatment |
| Shandong | Dermatology and Venereology | 0 | 5 | Know Treatment |
| Shandong | Oncology | 0.428571428571429 | 7 | Know Treatment |
| Shanghai | Oncology | 0.5 | 10 | Know Treatment |
| Shanghai | Pathology | 0 | 5 | Know Treatment |
| Shanxi | Oncology | 0.305555555555556 | 36 | Know Treatment |
| Shanxi | Pathology | 0.4 | 5 | Know Treatment |
| Sichuan | Internal Medicine | 0.0769230769230769 | 13 | Know Treatment |
| Sichuan | Oncology | 0.263157894736842 | 19 | Know Treatment |
| Tianjin | Internal Medicine | 0.294117647058824 | 17 | Know Treatment |
| Tibet | Anesthesiology | 0 | 5 | Know Treatment |
| Tibet | Emergency Medicine | 0.285714285714286 | 7 | Know Treatment |
| Tibet | Internal Medicine | 0.111111111111111 | 9 | Know Treatment |
| Tibet | Surgery | 0 | 7 | Know Treatment |
| Xinjiang | Internal Medicine | 0.333333333333333 | 6 | Know Treatment |
| Zhejiang | Surgery | 0 | 6 | Know Treatment |
| Beijing | Internal Medicine | 0.25 | 8 | Know Prognosis |
| Beijing | Pathology | 0.6 | 5 | Know Prognosis |
| Beijing | Surgery | 0.25 | 8 | Know Prognosis |
| Chongqing | Administration/Logistics | 0.254901960784314 | 51 | Know Prognosis |
| Chongqing | Anesthesiology | 0.0909090909090909 | 11 | Know Prognosis |
| Chongqing | Dentistry | 0.142857142857143 | 7 | Know Prognosis |
| Chongqing | Dermatology and Venereology | 0 | 8 | Know Prognosis |
| Chongqing | Emergency Medicine | 0.0416666666666667 | 24 | Know Prognosis |
| Chongqing | General Practice | 0.106382978723404 | 47 | Know Prognosis |
| Chongqing | Internal Medicine | 0.185314685314685 | 286 | Know Prognosis |
| Chongqing | Laboratory Medicine | 0 | 9 | Know Prognosis |
| Chongqing | Medical Imaging | 0.2 | 30 | Know Prognosis |
| Chongqing | Obstetrics and Gynecology | 0.09375 | 32 | Know Prognosis |
| Chongqing | Oncology | 0.542857142857143 | 35 | Know Prognosis |
| Chongqing | Ophthalmology | 0 | 8 | Know Prognosis |
| Chongqing | Other Departments | 0.244444444444444 | 45 | Know Prognosis |
| Chongqing | Otolaryngology | 0.333333333333333 | 6 | Know Prognosis |
| Chongqing | Pathology | 0.8 | 5 | Know Prognosis |
| Chongqing | Pediatrics | 0.111111111111111 | 45 | Know Prognosis |
| Chongqing | Rehabilitation Medicine | 0.195121951219512 | 41 | Know Prognosis |
| Chongqing | Surgery | 0.283236994219653 | 173 | Know Prognosis |
| Chongqing | Traditional Chinese Medicine | 0.258064516129032 | 31 | Know Prognosis |
| Guangdong | Dermatology and Venereology | 0.181818181818182 | 11 | Know Prognosis |
| Guangdong | Internal Medicine | 0.166666666666667 | 6 | Know Prognosis |
| Guangdong | Pathology | 0.75 | 8 | Know Prognosis |
| Guangdong | Surgery | 0 | 5 | Know Prognosis |
| Guangdong | Traditional Chinese Medicine | 0 | 6 | Know Prognosis |
| Guangxi | Dermatology and Venereology | 0.119047619047619 | 42 | Know Prognosis |
| Guangxi | Surgery | 0 | 6 | Know Prognosis |
| Guizhou | Internal Medicine | 0.142857142857143 | 28 | Know Prognosis |
| Guizhou | Medical Imaging | 0.2 | 10 | Know Prognosis |
| Hainan | Dermatology and Venereology | 0 | 5 | Know Prognosis |
| Hainan | Oncology | 1 | 5 | Know Prognosis |
| Hebei | Emergency Medicine | 0.166666666666667 | 6 | Know Prognosis |
| Hebei | Internal Medicine | 0.136363636363636 | 44 | Know Prognosis |
| Hebei | Medical Imaging | 0.333333333333333 | 6 | Know Prognosis |
| Hebei | Oncology | 0.282608695652174 | 46 | Know Prognosis |
| Hebei | Other Departments | 0 | 5 | Know Prognosis |
| Hebei | Surgery | 0.04 | 25 | Know Prognosis |
| Heilongjiang | Dermatology and Venereology | 0.35 | 20 | Know Prognosis |
| Heilongjiang | Internal Medicine | 0.133333333333333 | 15 | Know Prognosis |
| Heilongjiang | Oncology | 0.39622641509434 | 53 | Know Prognosis |
| Heilongjiang | Surgery | 0.3 | 10 | Know Prognosis |
| Henan | Dermatology and Venereology | 0.129032258064516 | 62 | Know Prognosis |
| Henan | Internal Medicine | 0.113207547169811 | 53 | Know Prognosis |
| Henan | Medical Imaging | 0 | 9 | Know Prognosis |
| Henan | Obstetrics and Gynecology | 0 | 12 | Know Prognosis |
| Henan | Oncology | 0.6 | 15 | Know Prognosis |
| Henan | Ophthalmology | 0 | 7 | Know Prognosis |
| Henan | Other Departments | 0.125 | 8 | Know Prognosis |
| Henan | Surgery | 0.138888888888889 | 36 | Know Prognosis |
| Henan | Traditional Chinese Medicine | 0.214285714285714 | 14 | Know Prognosis |
| Hubei | Dermatology and Venereology | 0.125 | 8 | Know Prognosis |
| Hubei | Oncology | 0.333333333333333 | 9 | Know Prognosis |
| Hunan | Dentistry | 0.375 | 8 | Know Prognosis |
| Hunan | Dermatology and Venereology | 0.0555555555555556 | 18 | Know Prognosis |
| Hunan | Internal Medicine | 0.0384615384615385 | 26 | Know Prognosis |
| Hunan | Oncology | 0.25 | 24 | Know Prognosis |
| Hunan | Otolaryngology | 0.428571428571429 | 14 | Know Prognosis |
| Hunan | Surgery | 0.235294117647059 | 34 | Know Prognosis |
| Jilin | Dermatology and Venereology | 0 | 8 | Know Prognosis |
| Liaoning | Dermatology and Venereology | 0.0714285714285714 | 42 | Know Prognosis |
| Qinghai | Dermatology and Venereology | 0.304347826086957 | 23 | Know Prognosis |
| Qinghai | Oncology | 0.454545454545455 | 11 | Know Prognosis |
| Shaanxi | Dermatology and Venereology | 0.08 | 25 | Know Prognosis |
| Shaanxi | Oncology | 0.6 | 5 | Know Prognosis |
| Shaanxi | Surgery | 0.166666666666667 | 6 | Know Prognosis |
| Shandong | Dermatology and Venereology | 0 | 5 | Know Prognosis |
| Shandong | Oncology | 0.428571428571429 | 7 | Know Prognosis |
| Shanghai | Oncology | 0.6 | 10 | Know Prognosis |
| Shanghai | Pathology | 0.6 | 5 | Know Prognosis |
| Shanxi | Oncology | 0.416666666666667 | 36 | Know Prognosis |
| Shanxi | Pathology | 0.8 | 5 | Know Prognosis |
| Sichuan | Internal Medicine | 0.0769230769230769 | 13 | Know Prognosis |
| Sichuan | Oncology | 0.315789473684211 | 19 | Know Prognosis |
| Tianjin | Internal Medicine | 0.529411764705882 | 17 | Know Prognosis |
| Tibet | Anesthesiology | 0 | 5 | Know Prognosis |
| Tibet | Emergency Medicine | 0.142857142857143 | 7 | Know Prognosis |
| Tibet | Internal Medicine | 0.111111111111111 | 9 | Know Prognosis |
| Tibet | Surgery | 0 | 7 | Know Prognosis |
| Xinjiang | Internal Medicine | 0.666666666666667 | 6 | Know Prognosis |
| Zhejiang | Surgery | 0.166666666666667 | 6 | Know Prognosis |
| Beijing | Internal Medicine | 0.125 | 8 | Know Fusion |
| Beijing | Pathology | 0.6 | 5 | Know Fusion |
| Beijing | Surgery | 0.25 | 8 | Know Fusion |
| Chongqing | Administration/Logistics | 0.156862745098039 | 51 | Know Fusion |
| Chongqing | Anesthesiology | 0 | 11 | Know Fusion |
| Chongqing | Dentistry | 0.142857142857143 | 7 | Know Fusion |
| Chongqing | Dermatology and Venereology | 0 | 8 | Know Fusion |
| Chongqing | Emergency Medicine | 0.0416666666666667 | 24 | Know Fusion |
| Chongqing | General Practice | 0.0212765957446809 | 47 | Know Fusion |
| Chongqing | Internal Medicine | 0.0804195804195804 | 286 | Know Fusion |
| Chongqing | Laboratory Medicine | 0 | 9 | Know Fusion |
| Chongqing | Medical Imaging | 0.0333333333333333 | 30 | Know Fusion |
| Chongqing | Obstetrics and Gynecology | 0.03125 | 32 | Know Fusion |
| Chongqing | Oncology | 0.257142857142857 | 35 | Know Fusion |
| Chongqing | Ophthalmology | 0 | 8 | Know Fusion |
| Chongqing | Other Departments | 0.0666666666666667 | 45 | Know Fusion |
| Chongqing | Otolaryngology | 0.166666666666667 | 6 | Know Fusion |
| Chongqing | Pathology | 0.8 | 5 | Know Fusion |
| Chongqing | Pediatrics | 0.0666666666666667 | 45 | Know Fusion |
| Chongqing | Rehabilitation Medicine | 0.0487804878048781 | 41 | Know Fusion |
| Chongqing | Surgery | 0.104046242774566 | 173 | Know Fusion |
| Chongqing | Traditional Chinese Medicine | 0 | 31 | Know Fusion |
| Guangdong | Dermatology and Venereology | 0.0909090909090909 | 11 | Know Fusion |
| Guangdong | Internal Medicine | 0 | 6 | Know Fusion |
| Guangdong | Pathology | 0.625 | 8 | Know Fusion |
| Guangdong | Surgery | 0 | 5 | Know Fusion |
| Guangdong | Traditional Chinese Medicine | 0 | 6 | Know Fusion |
| Guangxi | Dermatology and Venereology | 0.0238095238095238 | 42 | Know Fusion |
| Guangxi | Surgery | 0 | 6 | Know Fusion |
| Guizhou | Internal Medicine | 0 | 28 | Know Fusion |
| Guizhou | Medical Imaging | 0 | 10 | Know Fusion |
| Hainan | Dermatology and Venereology | 0 | 5 | Know Fusion |
| Hainan | Oncology | 0 | 5 | Know Fusion |
| Hebei | Emergency Medicine | 0 | 6 | Know Fusion |
| Hebei | Internal Medicine | 0.0227272727272727 | 44 | Know Fusion |
| Hebei | Medical Imaging | 0.166666666666667 | 6 | Know Fusion |
| Hebei | Oncology | 0.173913043478261 | 46 | Know Fusion |
| Hebei | Other Departments | 0 | 5 | Know Fusion |
| Hebei | Surgery | 0 | 25 | Know Fusion |
| Heilongjiang | Dermatology and Venereology | 0.1 | 20 | Know Fusion |
| Heilongjiang | Internal Medicine | 0 | 15 | Know Fusion |
| Heilongjiang | Oncology | 0.264150943396226 | 53 | Know Fusion |
| Heilongjiang | Surgery | 0.1 | 10 | Know Fusion |
| Henan | Dermatology and Venereology | 0.0483870967741935 | 62 | Know Fusion |
| Henan | Internal Medicine | 0.0377358490566038 | 53 | Know Fusion |
| Henan | Medical Imaging | 0 | 9 | Know Fusion |
| Henan | Obstetrics and Gynecology | 0 | 12 | Know Fusion |
| Henan | Oncology | 0.466666666666667 | 15 | Know Fusion |
| Henan | Ophthalmology | 0 | 7 | Know Fusion |
| Henan | Other Departments | 0 | 8 | Know Fusion |
| Henan | Surgery | 0.111111111111111 | 36 | Know Fusion |
| Henan | Traditional Chinese Medicine | 0 | 14 | Know Fusion |
| Hubei | Dermatology and Venereology | 0 | 8 | Know Fusion |
| Hubei | Oncology | 0.333333333333333 | 9 | Know Fusion |
| Hunan | Dentistry | 0 | 8 | Know Fusion |
| Hunan | Dermatology and Venereology | 0 | 18 | Know Fusion |
| Hunan | Internal Medicine | 0.0384615384615385 | 26 | Know Fusion |
| Hunan | Oncology | 0.0833333333333333 | 24 | Know Fusion |
| Hunan | Otolaryngology | 0.142857142857143 | 14 | Know Fusion |
| Hunan | Surgery | 0.147058823529412 | 34 | Know Fusion |
| Jilin | Dermatology and Venereology | 0 | 8 | Know Fusion |
| Liaoning | Dermatology and Venereology | 0 | 42 | Know Fusion |
| Qinghai | Dermatology and Venereology | 0.0434782608695652 | 23 | Know Fusion |
| Qinghai | Oncology | 0.181818181818182 | 11 | Know Fusion |
| Shaanxi | Dermatology and Venereology | 0.08 | 25 | Know Fusion |
| Shaanxi | Oncology | 0.2 | 5 | Know Fusion |
| Shaanxi | Surgery | 0.166666666666667 | 6 | Know Fusion |
| Shandong | Dermatology and Venereology | 0 | 5 | Know Fusion |
| Shandong | Oncology | 0.285714285714286 | 7 | Know Fusion |
| Shanghai | Oncology | 0.3 | 10 | Know Fusion |
| Shanghai | Pathology | 0.6 | 5 | Know Fusion |
| Shanxi | Oncology | 0.305555555555556 | 36 | Know Fusion |
| Shanxi | Pathology | 0.8 | 5 | Know Fusion |
| Sichuan | Internal Medicine | 0.0769230769230769 | 13 | Know Fusion |
| Sichuan | Oncology | 0.0526315789473684 | 19 | Know Fusion |
| Tianjin | Internal Medicine | 0 | 17 | Know Fusion |
| Tibet | Anesthesiology | 0 | 5 | Know Fusion |
| Tibet | Emergency Medicine | 0 | 7 | Know Fusion |
| Tibet | Internal Medicine | 0.111111111111111 | 9 | Know Fusion |
| Tibet | Surgery | 0 | 7 | Know Fusion |
| Xinjiang | Internal Medicine | 0.166666666666667 | 6 | Know Fusion |
| Zhejiang | Surgery | 0.166666666666667 | 6 | Know Fusion |
| Beijing | Internal Medicine | 0.625 | 8 | Know None |
| Beijing | Pathology | 0.2 | 5 | Know None |
| Beijing | Surgery | 0.75 | 8 | Know None |
| Chongqing | Administration/Logistics | 0.666666666666667 | 51 | Know None |
| Chongqing | Anesthesiology | 0.727272727272727 | 11 | Know None |
| Chongqing | Dentistry | 0.857142857142857 | 7 | Know None |
| Chongqing | Dermatology and Venereology | 0.625 | 8 | Know None |
| Chongqing | Emergency Medicine | 0.833333333333333 | 24 | Know None |
| Chongqing | General Practice | 0.787234042553192 | 47 | Know None |
| Chongqing | Internal Medicine | 0.72027972027972 | 286 | Know None |
| Chongqing | Laboratory Medicine | 0.777777777777778 | 9 | Know None |
| Chongqing | Medical Imaging | 0.7 | 30 | Know None |
| Chongqing | Obstetrics and Gynecology | 0.78125 | 32 | Know None |
| Chongqing | Oncology | 0.285714285714286 | 35 | Know None |
| Chongqing | Ophthalmology | 1 | 8 | Know None |
| Chongqing | Other Departments | 0.644444444444444 | 45 | Know None |
| Chongqing | Otolaryngology | 0.666666666666667 | 6 | Know None |
| Chongqing | Pathology | 0.2 | 5 | Know None |
| Chongqing | Pediatrics | 0.777777777777778 | 45 | Know None |
| Chongqing | Rehabilitation Medicine | 0.75609756097561 | 41 | Know None |
| Chongqing | Surgery | 0.572254335260116 | 173 | Know None |
| Chongqing | Traditional Chinese Medicine | 0.67741935483871 | 31 | Know None |
| Guangdong | Dermatology and Venereology | 0.818181818181818 | 11 | Know None |
| Guangdong | Internal Medicine | 0.666666666666667 | 6 | Know None |
| Guangdong | Pathology | 0 | 8 | Know None |
| Guangdong | Surgery | 1 | 5 | Know None |
| Guangdong | Traditional Chinese Medicine | 1 | 6 | Know None |
| Guangxi | Dermatology and Venereology | 0.785714285714286 | 42 | Know None |
| Guangxi | Surgery | 0.833333333333333 | 6 | Know None |
| Guizhou | Internal Medicine | 0.678571428571429 | 28 | Know None |
| Guizhou | Medical Imaging | 0.6 | 10 | Know None |
| Hainan | Dermatology and Venereology | 0.8 | 5 | Know None |
| Hainan | Oncology | 0 | 5 | Know None |
| Hebei | Emergency Medicine | 0.666666666666667 | 6 | Know None |
| Hebei | Internal Medicine | 0.772727272727273 | 44 | Know None |
| Hebei | Medical Imaging | 0.666666666666667 | 6 | Know None |
| Hebei | Oncology | 0.543478260869565 | 46 | Know None |
| Hebei | Other Departments | 0.8 | 5 | Know None |
| Hebei | Surgery | 0.88 | 25 | Know None |
| Heilongjiang | Dermatology and Venereology | 0.45 | 20 | Know None |
| Heilongjiang | Internal Medicine | 0.733333333333333 | 15 | Know None |
| Heilongjiang | Oncology | 0.490566037735849 | 53 | Know None |
| Heilongjiang | Surgery | 0.6 | 10 | Know None |
| Henan | Dermatology and Venereology | 0.580645161290323 | 62 | Know None |
| Henan | Internal Medicine | 0.773584905660377 | 53 | Know None |
| Henan | Medical Imaging | 0.777777777777778 | 9 | Know None |
| Henan | Obstetrics and Gynecology | 1 | 12 | Know None |
| Henan | Oncology | 0.266666666666667 | 15 | Know None |
| Henan | Ophthalmology | 1 | 7 | Know None |
| Henan | Other Departments | 0.75 | 8 | Know None |
| Henan | Surgery | 0.75 | 36 | Know None |
| Henan | Traditional Chinese Medicine | 0.714285714285714 | 14 | Know None |
| Hubei | Dermatology and Venereology | 0.75 | 8 | Know None |
| Hubei | Oncology | 0.444444444444444 | 9 | Know None |
| Hunan | Dentistry | 0.5 | 8 | Know None |
| Hunan | Dermatology and Venereology | 0.888888888888889 | 18 | Know None |
| Hunan | Internal Medicine | 0.923076923076923 | 26 | Know None |
| Hunan | Oncology | 0.666666666666667 | 24 | Know None |
| Hunan | Otolaryngology | 0.357142857142857 | 14 | Know None |
| Hunan | Surgery | 0.676470588235294 | 34 | Know None |
| Jilin | Dermatology and Venereology | 1 | 8 | Know None |
| Liaoning | Dermatology and Venereology | 0.738095238095238 | 42 | Know None |
| Qinghai | Dermatology and Venereology | 0.434782608695652 | 23 | Know None |
| Qinghai | Oncology | 0.363636363636364 | 11 | Know None |
| Shaanxi | Dermatology and Venereology | 0.68 | 25 | Know None |
| Shaanxi | Oncology | 0.4 | 5 | Know None |
| Shaanxi | Surgery | 0.833333333333333 | 6 | Know None |
| Shandong | Dermatology and Venereology | 1 | 5 | Know None |
| Shandong | Oncology | 0.428571428571429 | 7 | Know None |
| Shanghai | Oncology | 0.3 | 10 | Know None |
| Shanghai | Pathology | 0.2 | 5 | Know None |
| Shanxi | Oncology | 0.5 | 36 | Know None |
| Shanxi | Pathology | 0 | 5 | Know None |
| Sichuan | Internal Medicine | 0.846153846153846 | 13 | Know None |
| Sichuan | Oncology | 0.578947368421053 | 19 | Know None |
| Tianjin | Internal Medicine | 0.411764705882353 | 17 | Know None |
| Tibet | Anesthesiology | 1 | 5 | Know None |
| Tibet | Emergency Medicine | 0.285714285714286 | 7 | Know None |
| Tibet | Internal Medicine | 0.777777777777778 | 9 | Know None |
| Tibet | Surgery | 0.571428571428571 | 7 | Know None |
| Xinjiang | Internal Medicine | 0.166666666666667 | 6 | Know None |
| Zhejiang | Surgery | 0.5 | 6 | Know None |

Awareness rate refers to the proportion of respondents from each city and department who reported being familiar with a specific aspect of NUT carcinoma. “n” represents the number of respondents from each city and department for the corresponding knowledge aspect. “Knowledge” indicates the specific aspect of NUT carcinoma (clinical manifestations, diagnosis, treatment, prognosis, molecular fusion partners, or none) that the awareness rate refers to.

# Supplementary Table 5. Significant Factors Associated with Awareness of NUT Carcinoma Knowledge: Results from Multivariable Ridge Regression Analysise

| **DependentVariable** | **Variable** | **Beta** | **OR** | **CI_2.5** | **CI_97.5** | **StdErr** | **z** | **p_value** | **Significance** | **Adjusted_Alpha** |
| --- | --- | --- | --- | --- | --- | --- | --- | --- | --- | --- |
| Know Clinical | DepartmentOncology | 0.507767826504308 | 1.66157812173146 | 0.386977341619048 | 0.651775295199279 | 0.0704568520520358 | 7.20679127317947 | 5.72875080706581e-13 | *** | 9.25925925925926e-05 |
| Know Clinical | DepartmentOphthalmology | -1.036923 | 0.354543937079865 | -1.099455752 | -0.954185587 | 0.0354158826238789 | -29.27847403 | 0 | *** | 9.25925925925926e-05 |
| Know Diagnosis | CityXinjiang | 1.950786255 | 7.03421609701541 | 1.09443381259841 | 2.93415788291686 | 0.48120827041372 | 4.05393334924358 | 5.03635712252315e-05 | * | 9.25925925925926e-05 |
| Know Diagnosis | DepartmentOncology | 0.71497339381031 | 2.04413229496345 | 0.510707268856182 | 0.919966033961594 | 0.105282428982462 | 6.79100397588103 | 1.11355369369903e-11 | *** | 9.25925925925926e-05 |
| Know Diagnosis | DepartmentOphthalmology | -1.311131986 | 0.269514796766791 | -1.437260263 | -1.192055814 | 0.0569281558947109 | -23.03134477 | 0 | *** | 9.25925925925926e-05 |
| Know Diagnosis | DepartmentPathology | 2.23865976153958 | 9.38075041614682 | 1.85695471155018 | 2.58123044860006 | 0.19139659262827 | 11.6964452229695 | 0 | *** | 9.25925925925926e-05 |
| Know None | CityXinjiang | -1.947255397 | 0.142665093849661 | -2.572342828 | -1.010618311 | 0.448619292170181 | -4.340552068 | 1.42125184645625e-05 | ** | 9.25925925925926e-05 |
| Know None | DepartmentOncology | -0.661784713 | 0.515929726053644 | -0.837521105 | -0.459139059 | 0.0957841416967034 | -6.909126092 | 4.87654361336354e-12 | *** | 9.25925925925926e-05 |
| Know None | DepartmentOphthalmology | 1.56485723400689 | 4.78199218129655 | 1.46437530142635 | 1.67469126348676 | 0.0516840285806444 | 30.2773850448827 | 0 | *** | 9.25925925925926e-05 |
| Know None | DepartmentPathology | -1.918824395 | 0.14677941529016 | -2.29409048 | -1.575393918 | 0.178694518755143 | -10.73801485 | 0 | *** | 9.25925925925926e-05 |
| Know Prognosis | CityInner Mongolia | -0.92967614 | 0.394681511117504 | -1.082387142 | -0.728736239 | 0.0859568312070093 | -10.81561671 | 0 | *** | 9.25925925925926e-05 |
| Know Prognosis | DepartmentAnesthesiology | -0.832033642 | 0.435163419283547 | -1.140525877 | -0.393744651 | 0.202502927686079 | -4.108748705 | 3.9780864933503e-05 | * | 9.25925925925926e-05 |
| Know Prognosis | DepartmentObstetrics and Gynecology | -0.733533619 | 0.480209112544607 | -1.105488933 | -0.420907568 | 0.180135439271976 | -4.072122742 | 4.65866173307017e-05 | * | 9.25925925925926e-05 |
| Know Prognosis | DepartmentOncology | 0.799756551101658 | 2.22499918895083 | 0.59754386299053 | 1.0151583387622 | 0.0990325698783222 | 8.07569218979463 | 6.66133814775094e-16 | *** | 9.25925925925926e-05 |
| Know Prognosis | DepartmentOphthalmology | -1.127814252 | 0.323740097995938 | -1.258822208 | -1.020901109 | 0.0586927307436124 | -19.21556959 | 0 | *** | 9.25925925925926e-05 |
| Know Prognosis | DepartmentPathology | 1.54899546240173 | 4.70673971001578 | 1.08229254037474 | 1.97685978999277 | 0.21082104981109 | 7.34744212586805 | 2.02060590481778e-13 | *** | 9.25925925925926e-05 |
| Know Treatment | CityGansu | -0.832641453 | 0.434899002551967 | -0.983480339 | 0 | 0.20216700422136 | -4.118582338 | 3.81210360975714e-05 | * | 9.25925925925926e-05 |
| Know Treatment | CityJiangxi | -0.732153703 | 0.48087221809608 | -0.868329653 | -0.59043797 | 0.0661419947541429 | -11.06942277 | 0 | *** | 9.25925925925926e-05 |
| Know Treatment | CityJilin | -0.676444805 | 0.508421319784832 | -0.728534358 | -0.607426827 | 0.0309490916023517 | -21.85669338 | 0 | *** | 9.25925925925926e-05 |
| Know Treatment | CityLiaoning | -0.571282174 | 0.564800801425649 | -0.710873611 | -0.287875134 | 0.107082697167263 | -5.334962501 | 9.55643049138644e-08 | *** | 9.25925925925926e-05 |
| Know Treatment | DepartmentObstetrics and Gynecology | -0.46440069 | 0.628511665354549 | -0.668480623 | -0.214257953 | 0.117245903565691 | -3.960911864 | 7.46640896867046e-05 | * | 9.25925925925926e-05 |
| Know Treatment | DepartmentOncology | 0.564918236170909 | 1.75930392941509 | 0.413157706627326 | 0.734580464624288 | 0.0792374570752234 | 7.12943419719551 | 1.00786046175472e-12 | *** | 9.25925925925926e-05 |
| Know Treatment | DepartmentOphthalmology | -0.69859996 | 0.497281030170147 | -0.760372955 | -0.619011786 | 0.0329942641299641 | -21.17337598 | 0 | *** | 9.25925925925926e-05 |
| Know Clinical | DepartmentOncology | 0.489995967662726 | 1.63230963791911 | 0.326004233985978 | 0.649881995381017 | 0.0805662809336138 | 6.08189880412228 | 1.18767529144748e-09 | *** | 9.25925925925926e-05 |
| Know Clinical | DepartmentOphthalmology | -0.98737974 | 0.372551595427599 | -1.065412748 | -0.912795312 | 0.0360429532313479 | -27.39452933 | 0 | *** | 9.25925925925926e-05 |
| Know Diagnosis | DepartmentOncology | 0.698046594009488 | 2.00982287040155 | 0.501753891481356 | 0.896939896725773 | 0.0999050372120362 | 6.98710108608407 | 2.80619971704255e-12 | *** | 9.25925925925926e-05 |
| Know Diagnosis | DepartmentOphthalmology | -1.258090838 | 0.284196085158042 | -1.370102695 | -1.143816789 | 0.0503661488641031 | -24.97889687 | 0 | *** | 9.25925925925926e-05 |
| Know Diagnosis | DepartmentPathology | 2.18140835427334 | 8.85877376921872 | 1.80479705657704 | 2.47523532244772 | 0.169092322850183 | 12.9006942332094 | 0 | *** | 9.25925925925926e-05 |
| Know Prognosis | CityInner Mongolia | -0.890994679 | 0.410247485010334 | -1.069214516 | -0.714702123 | 0.0833798251154491 | -10.68597443 | 0 | *** | 9.25925925925926e-05 |
| Know Prognosis | DepartmentAnesthesiology | -0.800106386 | 0.449281164422996 | -1.072817139 | -0.256671403 | 0.202269345082391 | -3.955648274 | 7.63273897683714e-05 | * | 9.25925925925926e-05 |
| Know Prognosis | DepartmentObstetrics and Gynecology | -0.707381444 | 0.492933282387353 | -1.052469426 | -0.392531094 | 0.158098035373083 | -4.474321533 | 7.66543199559599e-06 | *** | 9.25925925925926e-05 |
| Know Prognosis | DepartmentOncology | 0.778743299093699 | 2.17873252920019 | 0.547635854543737 | 0.959610909677829 | 0.104696306849474 | 7.43811622900249 | 1.02140518265514e-13 | *** | 9.25925925925926e-05 |
| Know Prognosis | DepartmentOphthalmology | -1.078504269 | 0.340103849302064 | -1.191046198 | -0.9875845 | 0.0502751921475529 | -21.45201685 | 0 | *** | 9.25925925925926e-05 |
| Know Prognosis | DepartmentPathology | 1.51268912083845 | 4.53892010233896 | 1.08921489685717 | 1.89662210301617 | 0.20754347763439 | 7.28854087866455 | 3.13304937549219e-13 | *** | 9.25925925925926e-05 |
| Know Treatment | CityGansu | -0.933091912 | 0.39333566910645 | -1.081051154 | 0 | 0.207353094213351 | -4.500014409 | 6.79488563082309e-06 | *** | 9.25925925925926e-05 |
| Know Treatment | CityJiangxi | -0.817802988 | 0.441400351951496 | -0.950646981 | -0.675948208 | 0.0739490411970569 | -11.05900732 | 0 | *** | 9.25925925925926e-05 |
| Know Treatment | CityJilin | -0.754363591 | 0.470309828826965 | -0.862774781 | -0.673052565 | 0.0451833854160887 | -16.69559694 | 0 | *** | 9.25925925925926e-05 |
| Know Treatment | CityLiaoning | -0.632922499 | 0.531037574054103 | -0.784599084 | -0.408938751 | 0.105320905705256 | -6.009466925 | 1.86134330171228e-09 | *** | 9.25925925925926e-05 |
| Know Treatment | DepartmentOncology | 0.606180464629197 | 1.8334152137697 | 0.445983475628989 | 0.781646921016114 | 0.087743397590795 | 6.90855929076526 | 4.89608353859694e-12 | *** | 9.25925925925926e-05 |
| Know Treatment | DepartmentOphthalmology | -0.777595169 | 0.45950972702767 | -0.852288284 | -0.701593941 | 0.0387457683581877 | -20.06916372 | 0 | *** | 9.25925925925926e-05 |
| org A | CityInner Mongolia | -0.804585514 | 0.447273276820618 | -0.894505168 | -0.707683002 | 0.048052837538579 | -16.74376696 | 0 | *** | 9.25925925925926e-05 |
| org A | CityTianjin | -0.611791127 | 0.542378529882726 | -0.872791679 | -0.295376693 | 0.148846459029657 | -4.110216196 | 3.95288843733077e-05 | * | 9.25925925925926e-05 |
| org A | CityYunnan | -0.957388335 | 0.383894180786617 | -1.146071358 | 0 | 0.228534041207075 | -4.189259203 | 2.79866580814581e-05 | * | 9.25925925925926e-05 |
| org A | DepartmentOncology | 0.513686832942047 | 1.67144217723619 | 0.363542591119897 | 0.64819868218733 | 0.0727193005704801 | 7.06396828506591 | 1.6180390360887e-12 | *** | 9.25925925925926e-05 |
| org A | DepartmentOphthalmology | -0.715268199 | 0.489060928389127 | -0.91331176 | -0.360347961 | 0.155021611684813 | -4.613990214 | 3.95011030396297e-06 | *** | 9.25925925925926e-05 |
| org B | CityGansu | -0.433213206 | 0.648422229846238 | -0.501201854 | 0 | 0.106861165280807 | -4.053981673 | 5.03531630493548e-05 | * | 9.25925925925926e-05 |
| org B | CityInner Mongolia | -0.351929054 | 0.703330018818921 | -0.41434864 | -0.303078534 | 0.0275347619545926 | -12.78126371 | 0 | *** | 9.25925925925926e-05 |
| org B | CityNingxia | -0.387554675 | 0.67871452471009 | -0.461230554 | -0.309535293 | 0.0379239389358287 | -10.21926218 | 0 | *** | 9.25925925925926e-05 |
| org B | CityTianjin | -0.379352426 | 0.684304403445047 | -0.423792428 | -0.342832985 | 0.0216094869305961 | -17.55490203 | 0 | *** | 9.25925925925926e-05 |
| org B | CityYunnan | -0.419057354 | 0.657666474475032 | -0.539186369 | 0 | 0.0967517020347384 | -4.331265963 | 1.4825447357536e-05 | ** | 9.25925925925926e-05 |
| org B | DepartmentOphthalmology | -0.385715698 | 0.679963813149721 | -0.430222112 | -0.341570695 | 0.0224232011671296 | -17.20163394 | 0 | *** | 9.25925925925926e-05 |
| org C | CityGansu | -0.256200378 | 0.773986863535223 | -0.290736659 | 0 | 0.0634999016547638 | -4.034657866 | 5.46819158553369e-05 | * | 9.25925925925926e-05 |
| org C | CityInner Mongolia | -0.225497422 | 0.798119117953607 | -0.255480996 | -0.191810532 | 0.0157884086253436 | -14.2824668 | 0 | *** | 9.25925925925926e-05 |
| org C | CityJiangsu | -0.24341888 | 0.783943066776939 | -0.271296354 | -0.205489673 | 0.015473230082877 | -15.73161382 | 0 | *** | 9.25925925925926e-05 |
| org C | CityJiangxi | -0.23478244 | 0.790742864867121 | -0.261650708 | -0.202726421 | 0.0155435009947394 | -15.10486216 | 0 | *** | 9.25925925925926e-05 |
| org C | CityNingxia | -0.236779299 | 0.789165438720576 | -0.271787021 | -0.197253194 | 0.0294484164617579 | -8.040476435 | 8.88178419700125e-16 | *** | 9.25925925925926e-05 |
| org C | CityYunnan | -0.255025886 | 0.774896438413737 | -0.302298545 | 0 | 0.0519912593201168 | -4.905168479 | 9.33471796304985e-07 | *** | 9.25925925925926e-05 |
| org C | DepartmentAnesthesiology | -0.242298646 | 0.784821758849294 | -0.269568606 | -0.209516834 | 0.0148597845181702 | -16.30566348 | 0 | *** | 9.25925925925926e-05 |
| org C | DepartmentOphthalmology | -0.241720007 | 0.785276018262753 | -0.272661613 | -0.208375146 | 0.0151320903845986 | -15.97399971 | 0 | *** | 9.25925925925926e-05 |
| org none | CityInner Mongolia | 0.799395030083038 | 2.22419495036095 | 0.699478629495758 | 0.883008061517894 | 0.0715596592725188 | 11.1710290156458 | 0 | *** | 9.25925925925926e-05 |
| org none | CityYunnan | 0.954025431398171 | 2.59613923384993 | 0 | 1.12258327530713 | 0.242070649372055 | 3.94110328481774 | 8.11076904720931e-05 | * | 9.25925925925926e-05 |
| org none | DepartmentOncology | -0.518444491 | 0.595446049377629 | -0.645548731 | -0.369363068 | 0.067461126433977 | -7.685085009 | 1.53210777398272e-14 | *** | 9.25925925925926e-05 |
| org none | DepartmentOphthalmology | 0.718379383124297 | 2.05110645793662 | 0.45279899662223 | 0.908688916744732 | 0.130399956810542 | 5.50904617375013 | 3.60783269925946e-08 | *** | 9.25925925925926e-05 |
| org A | CityChongqing | 0.176951918106707 | 1.19357370246402 | 0.0807767287670394 | 0.259214776827195 | 0.0416397636381574 | 4.24958987866477 | 2.14162265419482e-05 | * | 9.25925925925926e-05 |
| org A | CityInner Mongolia | -0.888145966 | 0.41141782859476 | -0.990931782 | -0.772472687 | 0.0560673842121049 | -15.84068846 | 0 | *** | 9.25925925925926e-05 |
| org A | DepartmentOncology | 0.556918265788441 | 1.74528569756907 | 0.401706558261368 | 0.711229943263913 | 0.0764584406969907 | 7.28393439248311 | 3.24185123190546e-13 | *** | 9.25925925925926e-05 |
| org A | DepartmentOphthalmology | -0.78839927 | 0.454571860234131 | -1.010740133 | -0.420810534 | 0.161051588561531 | -4.895321285 | 9.81452491277679e-07 | *** | 9.25925925925926e-05 |
| org B | CityGansu | -0.533728913 | 0.586414200304014 | -0.609372899 | 0 | 0.117460433809642 | -4.543903809 | 5.52218648142144e-06 | *** | 9.25925925925926e-05 |
| org B | CityInner Mongolia | -0.422171468 | 0.655621611534582 | -0.482907681 | -0.339508823 | 0.036643818874907 | -11.52094626 | 0 | *** | 9.25925925925926e-05 |
| org B | CityNingxia | -0.4723046 | 0.623563546302518 | -0.58515329 | -0.37400552 | 0.0470841177879195 | -10.03108102 | 0 | *** | 9.25925925925926e-05 |
| org B | CityTianjin | -0.458443377 | 0.632267081473346 | -0.510312888 | -0.407542347 | 0.0233625804584692 | -19.62297691 | 0 | *** | 9.25925925925926e-05 |
| org B | DepartmentOphthalmology | -0.467848183 | 0.626348606531022 | -0.524731186 | -0.409297491 | 0.028211110907382 | -16.58382702 | 0 | *** | 9.25925925925926e-05 |
| org C | CityGansu | -0.276976961 | 0.75807196185947 | -0.318474625 | 0 | 0.0629507271515034 | -4.399900905 | 1.08300322483323e-05 | ** | 9.25925925925926e-05 |
| org C | CityInner Mongolia | -0.241868227 | 0.785159633451275 | -0.278641082 | -0.214487104 | 0.016592637891882 | -14.57683997 | 0 | *** | 9.25925925925926e-05 |
| org C | CityJiangsu | -0.262345634 | 0.769245100654824 | -0.29530105 | -0.228127642 | 0.016601486073269 | -15.80253916 | 0 | *** | 9.25925925925926e-05 |
| org C | CityJiangxi | -0.252482565 | 0.776869757395859 | -0.287994926 | -0.22047025 | 0.0243834448559211 | -10.35467165 | 0 | *** | 9.25925925925926e-05 |
| org C | CityNingxia | -0.254860724 | 0.775024433062206 | -0.3043111 | -0.212293664 | 0.0205940542811925 | -12.37545168 | 0 | *** | 9.25925925925926e-05 |
| org C | CityYunnan | -0.275653615 | 0.759075817861988 | -0.325009471 | 0 | 0.0610897851003988 | -4.512270167 | 6.41374185650356e-06 | *** | 9.25925925925926e-05 |
| org C | DepartmentAnesthesiology | -0.260920076 | 0.770342485634761 | -0.291527003 | -0.230349172 | 0.0156666857320202 | -16.6544527 | 0 | *** | 9.25925925925926e-05 |
| org C | DepartmentOphthalmology | -0.260315708 | 0.770808196885286 | -0.291402092 | -0.226111424 | 0.0158756828063697 | -16.39713463 | 0 | *** | 9.25925925925926e-05 |

**Note:** Only statistically significant variables from the multivariable ridge regression analysis are presented (Bonferroni-adjusted significance threshold α=9.26×10⁻⁵). Beta: regression coefficient; OR: odds ratio; CI_2.5 and CI_97.5: lower and upper bounds of the 95% confidence interval; StdErr: standard error; z: z-score; p_value: P value.“Know Clinical,” “Know Diagnosis,” “Know Prognosis,” “Know Treatment,” and “Know None” denote awareness of clinical, diagnostic, prognostic, treatment knowledge, and complete lack of knowledge about NUT carcinoma, respectively.Reference groups are the baseline categories for each variable (e.g., department, city).Ridge regression was used to address multicollinearity, and bootstrap (R=200) was applied for robust estimation of confidence intervals.Statistical significance: * P < 0.05, ** P < 0.01, *** P < 0.001 (all after Bonferroni correction).

# Supplementary Table 6. Model Performance Metrics of Multivariable Ridge Regression for Each Outcome Variable

| **Outcome Variable** | **Hosmer-Lemeshow P** | **AIC** | **Max VIF** | **AUC** | **Brier Score** |
| --- | --- | --- | --- | --- | --- |
| Know Clinical | 0.0015 | 2626.7 | 137.5 | 0.65 | 0.19 |
| Know Diagnosis | 0.0030 | 2339.7 | 112.5 | 0.70 | 0.16 |
| Know Treatment | 0.0001 | 2137.1 | 105.7 | 0.68 | 0.14 |
| Know Prognosis | 0.0059 | 2205.8 | 146.2 | 0.71 | 0.15 |
| Know Fusion | 0.0669 | 1272.7 | 84.5 | 0.79 | 0.07 |
| Know None | 0.0074 | 2732.8 | 134.3 | 0.68 | 0.20 |
| org A | 0.0001 | 2697.5 | 98.1 | 0.65 | 0.20 |
| org B | 0.00002 | 1505.1 | 65.5 | 0.68 | 0.09 |
| org C | 0.0002 | 1487.3 | 1.44E8 | 0.65 | 0.09 |
| org none | 0.000002 | 2742.1 | 100.0 | 0.65 | 0.20 |

**Note:** This table summarizes model evaluation metrics for each outcome in the multivariable ridge regression analysis, including the Hosmer–Lemeshow test P-value (for model calibration), Akaike Information Criterion (AIC, for model comparison), maximum Variance Inflation Factor (Max VIF, for multicollinearity assessment), Area Under the Receiver Operating Characteristic Curve (AUC, for discrimination), and Brier score (for prediction error). "org A", "org B", "org C", and "org none" refer to different outcomes related to organizational awareness.

# Supplementary Methods.Statistical analysis

After downloading and organizing the collected responses, descriptive statistics were performed to summarize participant characteristics, with categorical variables presented as frequencies and percentages. To systematically evaluate the impact of variables such as response time, city, specialty, and hospital level on NUT carcinoma knowledge and awareness of related organizations, multivariable ridge logistic regression models were applied.All independent variables were factorized as required, and hospital levels were recoded according to Chinese hospital classification standards. Binary outcome variables were rigorously screened and dichotomized to ensure model suitability.

Model development and optimization were performed using the glmnet package. Specifically, the optimal penalty parameter (lambda) was selected via five-fold cross-validation (nfolds = 5) using the cv.glmnet function to achieve the best generalization performance. Final ridge regression models were then fitted on the full dataset using the optimal lambda. To enhance the robustness and confidence of coefficient estimates, bootstrap resampling (R = 200) was conducted to calculate standard errors, p-values, and percentile-based confidence intervals for each parameter. All regression results are reported as odds ratios (ORs) and their 95% confidence intervals (exp(β) ± CI). The Bonferroni method was used to adjust significance levels for multiple comparisons, ensuring the rigor of statistical inference.

Variance inflation factors (VIFs) were calculated for each multivariable model to diagnose multicollinearity among independent variables; VIF > 10 was considered indicative of significant multicollinearity, and detailed VIF results were reported as needed. Model diagnostics and performance evaluation included area under the receiver operating characteristic curve (AUC, for discrimination), Brier score (for predictive accuracy), Hosmer-Lemeshow test (for calibration), Akaike Information Criterion (AIC), likelihood ratio test (LRT), and Wald test. Calibration curves were plotted using the val.prob function from the rms package, and all calibration figures were archived for reference.

All statistical analyses and visualizations were performed using R version 4.5, primarily utilizing the glmnet, boot, car, pROC, rms, and ggplot2 packages. Ridge regression was selected for its ability to improve the stability of estimates in the presence of multicollinearity, effectively control model complexity via L2 regularization, reduce parameter variance, and enhance both predictive accuracy and interpretability. Significant variables from all multivariable ridge regression models were visualized in forest plots (ORs and confidence intervals) to intuitively display the effects of influencing factors on knowledge awareness rates. All analytical procedures and parameter selections were documented to ensure reproducibility and scientific rigor.
